# Nanocellulose‐MXene Biomimetic Aerogels with Orientation‐Tunable Electromagnetic Interference Shielding Performance

**DOI:** 10.1002/advs.202000979

**Published:** 2020-06-28

**Authors:** Zhihui Zeng, Changxian Wang, Gilberto Siqueira, Daxin Han, Anja Huch, Sina Abdolhosseinzadeh, Jakob Heier, Frank Nüesch, Chuanfang (John) Zhang, Gustav Nyström

**Affiliations:** ^1^ Laboratory for Cellulose & Wood Materials Swiss Federal Laboratories for Materials Science and Technology (Empa) Dübendorf 8600 Switzerland; ^2^ School of Materials Science and Engineering Nanyang Technological University 50 Nanyang Avenue Singapore 639798 Singapore; ^3^ Department of Information Technology and Electrical Engineering Swiss Federal Institute of Technology in Zurich (ETH Zürich) Zürich 8092 Switzerland; ^4^ Laboratory for Functional Polymers Empa Dübendorf 8600 Switzerland; ^5^ Institute of Materials Science and Engineering Swiss Federal Institute of Technology Lausanne (EPFL) Lausanne 1015 Switzerland; ^6^ Department of Health Science and Technology ETH Zürich Schmelzbergstrasse 9 Zürich 8092 Switzerland

**Keywords:** aerogels, cellulose nanofibrils, EMI shielding, lightweight materials, MXenes

## Abstract

Designing lightweight nanostructured aerogels for high‐performance electromagnetic interference (EMI) shielding is crucial yet challenging. Ultrathin cellulose nanofibrils (CNFs) are employed for assisting in building ultralow‐density, robust, and highly flexible transition metal carbides and nitrides (MXenes) aerogels with oriented biomimetic cell walls. A significant influence of the angles between oriented cell walls and the incident EM wave electric field direction on the EMI shielding performance is revealed, providing an intriguing microstructure design strategy. MXene “bricks” bonded by CNF “mortars” of the nacre‐like cell walls induce high mechanical strength, electrical conductivity, and interfacial polarization, yielding the resultant MXene/CNF aerogels an ultrahigh EMI shielding performance. The EMI shielding effectiveness (SE) of the aerogels reaches 74.6 or 35.5 dB at a density of merely 8.0 or 1.5 mg cm^–3^, respectively. The normalized surface specific SE is up to 189 400 dB cm^2^ g^–1^, significantly exceeding that of other EMI shielding materials reported so far.

## Graphical abstract

Oriented biomimetic hybrid cell walls of nanocellulose‐MXene aerogels for tunable electromagnetic interference shielding and mechanism are represented by a yin‐yang symbol. Herein, the blue and red regions in the symbol correspond to angles with a smaller and larger transmission of the incident EM waves, respectively.

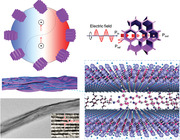

High‐performance electromagnetic interference (EMI) shielding materials with excellent EMI shielding effectiveness (SE) combined with low material consumption and good mechanical flexibility are emergently desirable for attenuating unwanted EM radiation or interference.^[^ ^1^ ^]^ Cellular architectures,^[^ ^2^ ^]^ consisting of nanostructured frame materials and micrometer‐sized pores, have shown a great potential for substituting the conventional metal shields due to their low density, considerable flexibility, easy processability, chemical stability, and tunable EMI shielding performance.^[^ ^3^ ^]^ Particularly, the existing pores enable multiple reflections (multi‐reflections) of incident EM waves in the shields, which greatly enlarge the intrinsic shielding ability of the frame materials.^[^ ^4^ ^]^ As such, designing the frame materials, the cellular microstructures, and their synergistic effects in these architectures for controllable EMI SE have always been a focus of research. Carbon and metal nanostructured porous scaffolds have been widely developed, such as carbon nanotube (CNT) sponges,^[^ ^5^ ^]^ polydimethylsiloxane‐coated graphene foams,^[^ ^6^ ^]^ and silver nanowire (Ag NW)‐embedded polymer aerogels.^[^ ^7^ ^]^ They can show SE values of around 20 dB, a commercial SE requirement,^[^ ^8^ ^]^ at low densities of 8–60 mg cm^–3^. The specific SE (SSE),^[^ ^6^, ^9^ ^]^ defined as SE divided by the density for evaluating lightweight EMI shielding architectures, reaches 333–5480 dB cm^3^ g^–1^, which is much higher than that (≈10 dB cm^3^ g^–1^) of solid copper or stainless steel at similar thicknesses.^[^ ^10^ ^]^ Moreover, the design of pore microstructure such as anisotropic microhoneycomb‐like cellular morphology has attracted increasing attention due to the vital influences on mechanical and functional attributes of the architectures.^[^ ^11^ ^]^ As such, the number of interfaces between the frame materials and voids is increased in one direction (transverse direction), further increasing the multi‐reflections and thus shielding ability of architectures.^[^ ^11^, ^12^ ^]^ Honeycomb‐like cellular architectures based on CNT, Ag NW, and graphene have been fabricated for EMI shielding applications.^[^ ^3^, ^11^, ^12^, ^13^ ^]^ Their SSE and thickness‐reduced SSE^[^ ^1^, ^3^, ^4^, ^11^ ^]^ (SSE/*t*, a normalized surface specific SE that is defined as SE divided by the density and thickness) can reach up to 9280 and 46 400 dB cm^2^ g^–1^, respectively. These values, however, are still unsatisfactory due to the limited utilization of the frame materials and cellular structures. Moreover, employing the anisotropic structures directly for the EM shielding results in a poor SE controllability, requiring precise sample dimensions to reach the target shielding functionality. In other words, designing efficient microstructure and understanding its unique interactions with EM waves are of critical importance, yet remain largely unexplored, in order to achieve excellent shielding performance of the nanostructured aerogels.

Recently, a new class of 2D metal carbides and nitrides, so‐called MXenes, have been quickly attracting tremendous attention due to their exotic properties, such as metallic conductivity, excellent mechanical properties, and large specific surface area.^[^ ^14^ ^]^ Owing to the selective removal of the A atomic layer from the parental MAX phase (where M is transition metal, A is group 13 or 14 element, X is carbon and/or nitrogen), MXenes are terminated with surface functional groups (—F, —OH, and —O), resulting in easy processability from their aqueous dispersion. This provides a great potential for the construction of high EMI shielding performance MXene‐based architectures.^[^ ^1^, ^13^, ^15^ ^]^ For instance, when introducing cellular structures by hydrazine treatment, the resultant MXene foam (with a density ≈0.22–0.39 g cm^–3^) showcases much enhanced EMI SE, exhibiting a breakthrough SSE/*t* of 136 752 dB cm^2^ g^–1^ even at a high EMI SE of 32 dB.^[^ ^4a^ ^]^ MXene aerogels (ultralow density ≈6 mg cm^–3^) prepared from multilayered MXene aqueous suspension also display a SSE/*t* of 49 520 dB cm^2^ g^–1^ at a high EMI SE of 60 dB.^[^ ^16^ ^]^ Nevertheless, the weak gelation capability and the tendency of MXenes to stack or aggregate limits the assembling of nanosheets into low‐density yet robust aerogels.^[^ ^13^, ^17^ ^]^ Some additives with strong self‐gelation (such as graphene oxide (GO), and polymer matrix/binders) are typically added to strengthen the mechanical properties in the resultant MXene‐based 3D framework, in spite of their increased density^[^ ^18^ ^]^ (24–44 mg cm^–3^ in the MXene/GO composites^[^ ^13b^ ^]^) or significantly compromised electronic conductivity.^[^ ^19^ ^]^ In other words, designing lightweight (particularly in the ultralow density range^[^ ^20^ ^]^ below 10 mg cm^–3^) yet robust and flexible MXene‐based hybrids with good conductivity and excellent EMI shielding performance has proved to be quite challenging.

Herein, we resolve the abovementioned issues by utilizing 1D cellulose nanofibrils (CNFs)^[^ ^2^, ^11^, ^14^, ^21^ ^]^ to assist in the fabrication of ultralow‐density MXene aerogels through an ice‐templated freeze‐casting approach.^[^ ^22^ ^]^ The CNFs are capable to well‐disperse/stabilize the large MXene monolayers, and bind the 2D nanosheets into high‐strength nacre‐like^[^ ^15^, ^23^ ^]^ MXene/CNF hybrid cell walls in a microhoneycomb‐like 3D architecture. The addition of thin CNFs (≈1.4 nm diameter) among the 2D layers also improves the interfacial polarization between MXenes and CNFs while preserving high conductivity. As a result, conductive MXene/CNF hybrid aerogels with ultralow densities yet high robustness and flexibility have been fabricated. The hybrid aerogels showcase EMI SE up to 74.6 dB, SSE as high as 30 660 dB cm^3^ g^–1^, and SSE/*t* achieving 189 400 dB cm^2^ g^–1^, exceeding that of other MXene‐based or other shielding architectures reported so far. In particular, we reveal that the presence of highly oriented biomimetic cell walls effectively governs the EMI shielding performance through an adjustment of their orientation angle to the electric field direction of the incident EM waves. This new phenomenon agrees well with the finite element analysis (FEA) simulation results and allows us to optimize EMI shielding performance without tuning the frame materials in the aerogels.

We begin with the MXene synthesis and delamination (Figure S1a, Supporting Information). To reduce the defect density on the MXene nanosheets, we first employed an etching method less aggressive than convential methods, the so‐called minimally intensive layer delamination to produce multilayered (m‐) Ti_3_C_2_T*_x_*. After etching, the Ti_3_AlC_2_ MAX precursors, with a compact rock‐like morphology structure, changed to m‐Ti_3_C_2_T*_x_* with a certain degree of delamination, best seen in the scanning electron microscopy (SEM) images (Figure S1b,c, Supporting Information). Upon repeated washing with deionized water to allow the ion exchange with the pre‐intercalated Li^+^, followed by vigorous manual shaking to further swell the clay‐like Ti_3_C_2_T*_x_*, a viscous aqueous ink enriched with delaminated Ti_3_C_2_T*_x_* MXene nanosheets was finally obtained (Figure S1d–g, Supporting Information). The X‐ray diffraction (XRD) pattern as well as Raman spectrum further verify the successful etching and delamination. The MXene viscous ink (Figure S1g, Supporting Information, concentration ≈30 mg mL^–1^) was made of clean flakes with a hexagonal atomic structure, according to transmission electron microscopy (TEM) images and electron diffraction (**Figure** **1**a and inset). The atomic force microscopy (AFM) analysis further confirms that the delaminated nanosheets are clean and composed of 71% monolayered, 16% bilayered, and 10% trilayered nanosheets (Figure 1b; Figure S1h–j, Supporting Information). Monolayer flakes are ≈1.5 nm in thickness and 3.2 ± 1.2 µm in mean lateral dimension, according to the AFM height profiles and statistics, respectively, and agreeing with our previous reports.^[^ ^14^, ^24^ ^]^

**Figure 1:**
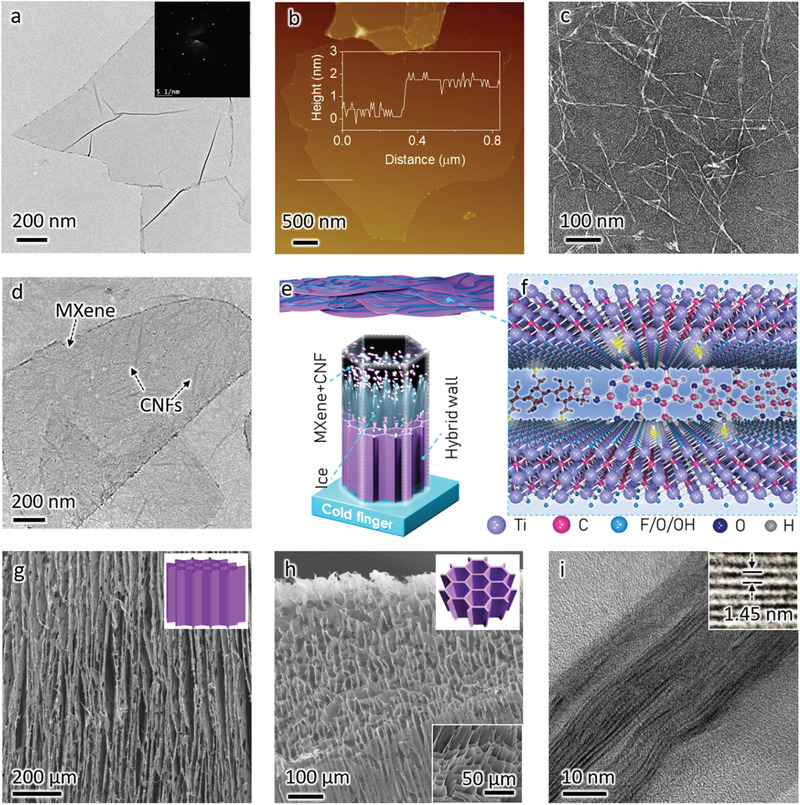
The structure and preparation process of the MXene/CNF biomimetic hybrid aerogel. a) TEM image and b) AFM image (the inset shows the edge profile of a monolayer) of the as‐prepared, large‐area monolyer MXene. c) TEM image of the as‐prepared CNFs with thin dimension and large aspect ratio. d) TEM image of the MXene layer with attached CNFs, showing the strong attraction and adhesion between the MXenes and CNFs. e) Schematic of the assemblied MXene/CNF hybrid cell walls and the unidirectional freezing process of the MXene/CNF‐mixed dispersion. f) Schematic of the MXene, the CNFs, and their noncovalent interactions. SEM image of the g) longitudinal plane and h) transverse plane for the MXene/CNF hybrid aerogels with 17 wt% CNF and density of 4 mg cm^–3^, and i) cross‐sectional TEM images of the corresponding MXene/CNF hybrid cell walls.

Stable CNF dispersion was prepared by 2,2,6,6‐tetramethylpiperidine‐1‐oxyl (TEMPO)‐mediated oxidation of the biomass cellulose fibers,^[^ ^2^, ^14^, ^25^ ^]^ followed by grinding to weaken the hydrogen bonds between neighboring cellulose chains (Figure S2a–g, Supporting Information). The transmission electron microscopy (TEM, Figure 1c) and AFM image (Figure S2h,i, Supporting Information) indicate that the as‐prepared CNFs own average ≈450 nm in length and ≈1.4 nm in diameter, suggesting a large aspect ratio (≈321). These CNFs are terminated with abundant hydrophilic, negatively charged surface groups (i.e., —OH and —COOH), resulting in a high zeta potential (−60 mV) and low viscosity of the stable aqueous dispersion (Figure S2j, Supporting Information). This enables homogeneous mixing of MXene and CNF aqueous dispersions without additional surfactants, best evidenced by the Tyndall effect and zeta potential (Figure S3, Supporting Information). The similarities in hydrophilicity and surface groups render a uniform deposition/adhesion of CNFs on the MXene nanosheets (Figure 1d), allowing the former to work as efficient structural directing agent^[^ ^14^, ^25^ ^]^ in the aerogel fabrication. Figure 1e shows the scheme of preparation of the anisotropic honeycomb‐like MXene/CNF hybrid aerogels, where the unidirectional ice‐templated freeze‐drying method was employed. During synthesis, the as‐formed pyramid‐like ice crystals exclude the CNF‐attached MXene layers on the surface, leading to the formation of oriented, interconnected MXene hybrid cell walls. The assembly of the intact cell walls can be interpreted as the CNF “nanomortars” efficiently crosslinking the MXene “nanobricks”^[^ ^15^, ^23^ ^]^ through the hydroxyl‐containing groups (Figure 1e,f), which eventually contribute to the formation of robust hybrid aerogels even at a density of 4 mg cm^–3^. The electron microscopy images further confirm our analysis. Oriented cell walls and unidirectional pore channels with gap ≈20 µm are clearly observed in the longitudinal plane (Figure 1g); the structure tends to be isotropic in the transverse plane of the MXene/CNF hybird aerogels (Figure 1h). A close examination of a typical hybrid aerogel (17 wt% CNF, density ≈4 mg cm^–3^) reveals that the biomimetic cell walls possess an average thickness of ≈36 nm with the gap among the stacked layers of ≈1.45 nm (Figure 1i; Figure S4, Supporting Information).

The cell walls' thickness is solution concentration dependant while the pore channels' diameter is influenced by the induced temperature gradient. As a result, nearly identical pore gap beween adjacent cell walls and similar cell walls' thickness have been achieved in the hybrid aerogels regardless of the CNF content (**Figure** **2**a; Figure S5a, Supporting Information). When CNF is absent, the pure MXene aerogels showcase a loose, leaves‐like morphology with abundant holey cell walls due to the weak gelation ability of MXene nanosheets (Figure 2b; Figure S5b, Supporting Information). The presence of the holes greatly compromises the mechanical properties in the resultant aerogels, hindering their large‐area preparation. In contrast, ultralight, freestanding, large‐area (12 cm × 6 cm), highly flexible (i.e., bendable and rollable), and easy‐shapable MXene/CNF hybrid aerogels can be fabricated (Figure 2c; Figures S6 and S7, Supporting Information), showing a great potential of these aerogels prepared through the easy‐to‐scale‐up ice‐templated approach for high‐performance EMI shielding application, as discussed below. By utilizing the CNF “mortars” to improve the gelation among the “nanobricks,” one can effectively enhance the compressive strength and modulus of the anisotropic hybrid aerogels, depending on the CNF content (Figure 2d; Figure S8, Supporting Information). By adding 33 wt% CNFs, the compressive modulus of the hybrid aerogel is up to 9.61 kPa, reaching ≈350% higher values than the modulus of pure MXene aerogels, highlighting the critical role of CNFs in the fabrication of ultralight yet robust aerogels.

**Figure 2:**
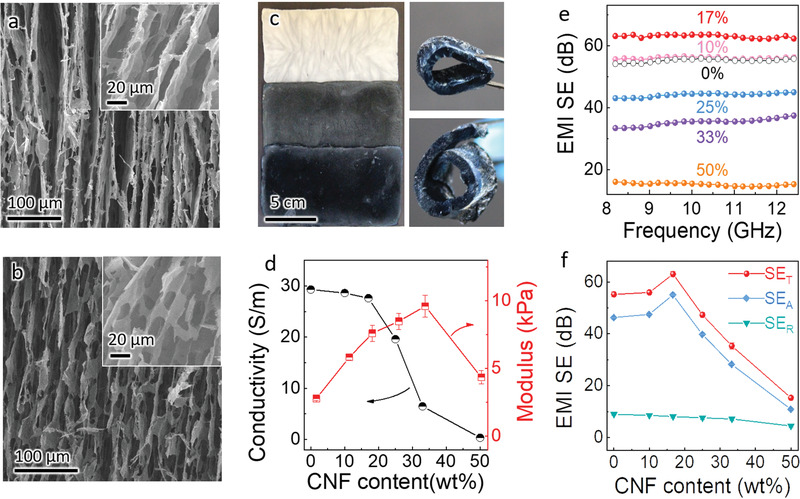
Structure and properties of the MXene/CNF hybrid aerogels with various CNF contents. Microstructures of the MXene/CNF hybrid aerogels with a) 17 wt% and b) pure MXene aerogels, at densities of 4 mg cm^–3^. c) Optical images of the MXene‐based hybrid aerogels, showing large‐area MXene/CNF aerogels (12 × 6 cm^2^) with various CNF contents (from top to bottom, corresponding to the pure CNF aerogels, the MXene/CNF with 50 wt% CNF, and MXene/CNF with 17 wt% CNF) at a density of 4 mg cm^–3^, and an image demonstrating the flexible performance of the MXene/CNF aerogels with 17 wt% CNF. d) Electrical conductivity and compressive modulus of the MXene‐based aerogels (4 mg cm^–3^) with various CNF contents. e) EMI SE in the X‐band and f) shielding performance at 10 GHz of the MXene‐based hybrid aerogels (4.0 mg cm^–3^) with various CNF contents.

Actually, the CNF content affects the microstructures and electrical properties of the resultant hybrid aerogels as well (Figure 2d; Table S1, Supporting Information). According to the XRD patterns (Figure S9a, Supporting Information), increasing the CNF content up to 17 wt% leads to a gradual downshift of the (002) characteristic peak of MXene, indicative of slightly broadened interlayer spacing from 13.97 to 14.84 Å that agrees well with the value obtained from the high‐resolution TEM (inset of Figure 1i). This ultrathin insulating phase between MXene nanosheets is beneficial for maintaining the high conductivity of the MXene‐based hybrid aerogels, e.g., a CNF content of 10 or 17 wt% results in a nearly identical electrical conductivity to that of pure MXene aerogels. Further increasing the amount of CNF results in a larger interlayer spacing, i.e., 15.63 Å when adding 50% of CNF, and the electrical conductivity of the resultant hybrid aerogel observably reduces. This is instructive as a small enlargement of interlayer spacing (6%) can increase the utilization of readily accessible nanosheets for the incident EM waves without compromising the electrical conductivity of the aerogel. Fourier transform infrared (FTIR) spectra of the aerogels further show the successful introduction^[^ ^15^ ^]^ of CNF into the as‐prepared hybrid aerogel (Figure S9b, Supporting Information). In other words, the interactions of the CNFs and MXenes offer a great potential for high EMI shielding performance of the robust and conductive MXene/CNF hybrid aerogels at a low CNF content.

In general, EMI shielding performance is influenced by the reflection (SE_R_), absorption (SE_A_), and multi‐reflections, corresponding to the mobile charge carriers, electric dipoles, and interior interfaces/surfaces, respectively.^[^ ^1^, ^2^, ^4^, ^6^, ^11^ ^]^ The multi‐reflections of incident EM waves induce more interactions with the pore cells in the porous architectures, efficiently increasing wave‐absorption ability of the shields. Therefore, the SE_A_ in the aerogels can dominate the total SE (SE_T_), which sums up both SE_R_ and SE_A_. As mentioned before, the interactions between MXenes and a small fraction of CNFs (i.e., 17 wt%) efficiently enhance the intactness of the hybrid cell walls and introduce effective interfaces between CNFs and MXenes. The large mismatch of electrical conductivity in the interfaces (the conductivities of the pure MXene and CNF are ≈10^6^ and ≈10^−10^ S m^–1^, respectively) leads to high interfacial polarizations,^[^ ^7^, ^12^, ^26^ ^]^ which, in combination with the abundant charge carriers from MXenes, resulting in increased wave absorption ability of the cell walls. The hybrid aerogel can thus achieve maximum SE_T_ and SE_A_ of 63.1 and 55.0 dB, respectively, with increased CNF content up to 17 wt% (Figure 2e,f; Figure S10, Supporting Information). Excessive CNF fraction inevitably decreases the conductivity and reduces the number of charge carriers of the aerogels, which greatly compromises the EMI SE. For example, the MXene/50% CNF aerogel demonstrates SE_T_ and SE_A_ of 15.3 and 10.8 dB, respectively, at a similar density of 4.0 mg cm^–3^ and an identical thickness of 2 mm. Moreover, the low‐density aerogel (17 wt% CNF) can demonstrate excellent EMI SE stability, as the EMI SE keeps almost constant after bending for 1000 cycles (Figure S11, Supporting Information). Therefore, we chose 17 wt% CNF as the optimized content to fabricate the hybrid aerogels unless specifically noted.

In this work, the EMI shielding measurements were performed as the incident EM waves propagate along the transverse direction, i.e., perpendicular to the direction of the pore channels (**Figure** **3**a). As such, more void‐cell wall interfaces are utilized to generate more multi‐reflections of the waves and exhibit higher transverse SE_T_ and SE_A_ values than those achieved as the waves propagate along the longitudinal direction (namely along the pore channels) (Figure S12, Supporting Information). Based on this, we controlled the oritentation of the cell walls to induce various angles between the oriented cell walls and the electric field direction of the incident EM waves (Figure 3b,c). An angle of 0° is defined when the oriented cell walls/pore channels are parallel to the electric field direction. The EMI SE values of the hybrid aerogel (density of 4 mg cm^–3^) were measured, as shown in Figure 3d. Interestingly, the EMI SE reaches its maximum at the angle of 0° (63.1 dB), then gradually decreases to 36.5 dB in the whole frequency range when increasing the angle up to 90°.

**Figure 3:**
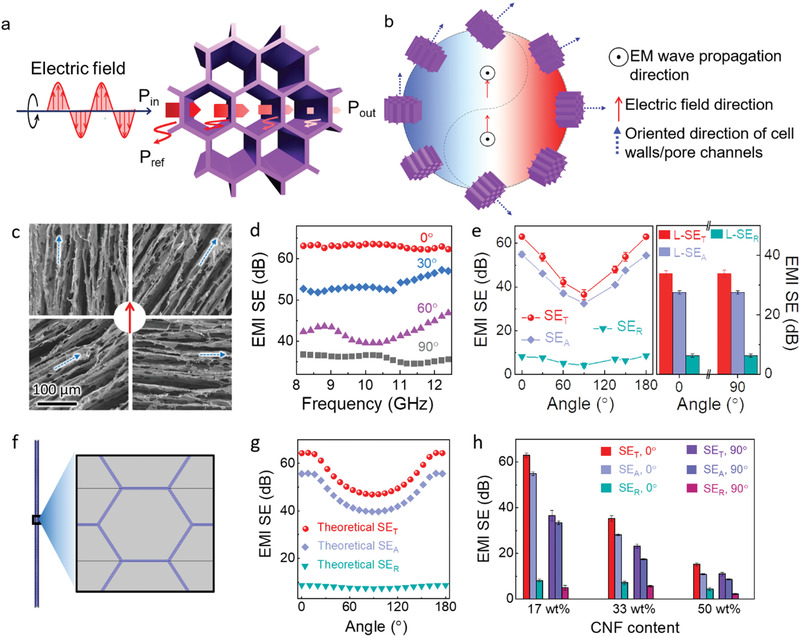
Oriented cell walls‐induced EMI shielding mechanism of the MXene/CNF hybrid aerogels. a) The EMI shielding mechanism when the incident EM waves propagate in the transverse direction, showing the most multi‐reflections of EM waves in this direction, and b) corresponding cell walls'/pore channels' orientation‐induced EMI shielding mechanism, which is reflected by the yin‐yang symbol that represents giving birth to or controlling everything in ancient China. Herein, the blue and red regions in the symbol correspond to angles with a smaller and larger transmission of the incident EM waves, respectively. c) SEM images of the MXene/CNF hybrid aerogels with various angles (0°, 30°, 60°, and 90°) between the cell walls' oriented direction and electric field direction of incident EM waves. d) EMI SE of the MXene/CNF hybrid aerogels with CNF content of 17 wt% and density of 4 mg cm^–3^ at various angles between the cell walls' oriented direction and electric field direction of incident EM waves, and the angle‐induced e) transverse (SE_T_, SE_A_, and SE_R_) and longitudinal (L‐SE_T_, L‐SE_A_, and L‐SE_R_) EMI shielding performance change at 10 GHz frequency. f) Theoretical simulation and g) calculation of the EMI shielding performance at 10 GHz for the honeycomb‐like porous architectures at various angles between the cell walls' oriented direction and electric field direction of incident EM waves. h) EMI shielding performance at a fixed frequency of 10 GHz for the MXene/CNF hybrid aerogels (4 mg cm^–3^) with various CNF contents at cell wall‐electric field angles of 0° and 90°, respectively.

To further clarify the EMI shielding behavior derived from the oriented cell walls/pore channels, we plotted the transverse SE_T_, SE_A_, and SE_R_ of the typical hybrid aerogel as a function of angles (Figure 3e). In the MXene/CNF hybrid cell walls, the presence of abundant mobile charge carriers and the dipoles arising from interfacial polarization and charge carriers jointly induce a strong interior electric field under the external EM field of incident waves. The internal electric field partially offsets the external EM field and transfers the dissipated EM energy to heat. At the angle of 0°, all cell walls in the aerogels are in parallel to the external electric field direction of incident EM waves, generating a strong internal electric field and maximum SE_T_ and SE_A_. Upon increasing the angle gradually from 0° to 90°, the number of cell walls that are in parallel to the external electric field direction is decreased and thus the intensity of internal electric field is reduced, corresponding to decreased EMI SE (SE_A_ and SE_R_). In contrast, the longitudinal shielding performance (L‐SE_T_, L‐SE_A_, and L‐SE_R_) does not illustrate a difference at different angles since the electric field direction is always perpendicular to the pore channels/cell walls. Further increasing the angle from 90° to 180° recovers the EMI SE gradually. For instance, the SE_T_ and SE_A_ bounce back to 63.0 and 54.5 dB, respectively, at an angle of 180°, which are nearly equal to the values achieved at angle of 0°. This is understandable, as the EMI SE is maximized (minimized) only if the oriented cell walls/pore channels of the aerogels are in parallel (perpendicular) to the electric field direction of incident EM waves.

To verify the above statements, FEA was carried out based on the parameters of the MXene/CNF hybrid aerogels to simulate the EMI shielding performance. Herein, the pore size and the thickness of cell walls were assumed as 20 µm and 36 nm, respectively, for the typical MXene/CNF aerogel (17 wt% CNF content, density ≈4.0 mg cm^–3^) (Figure 3f). The electrical conductivity of the cell walls was assumed the same as the measured value (50 000 S m^–1^) of solid MXene/CNF films (17 wt% CNF content) prepared by a cast‐drying approach. As demonstrated in Figure 3g and Figure S13a, Supporting Information, the theoretical simulation predicts an EMI SE_T_ value of 64.8 ± 2.6 dB in the X‐band at angle of 0°, quite close to the measured SE_T_ value. In addition, the numerically calculated EMI SE_T_ decreases first upon increasing the angle, and reaches 47.3±1.7 dB at an angle of 90° (Figure S13b, Supporting Information), beyond which SE_T_ starts to increase till an angle of 180° (Figure 3g). The numerical calculations (SE_T_, SE_A_, and SE_R_) agree quite well with the experimental results. It is worth noting that numerically simulated honeycomb‐like architectures are more ideal with fully interconnected highly conductive cell walls, as compared to the as‐preapared MXene/CNF hybrid aerogels. Still, the numerical SE was found to be similar to the experimental SE, although the numerical calculation ignores interfacial polarizations and MXene surface terminations that exert certain influences on the improved EMI shielding performance.^[^ ^4^, ^26^ ^]^ In short, both the experimental and theoretical results suggest that one can tune the EMI SE by simply adjusting the angle between the oriented pore channels/cell walls and the electric field direction of EM waves. Or to put it differently, it allows us to realize maximized EMI shielding performance in a given material architecture without altering either the sample dimension, or the constructed frame materials, or the incident wave propagation direction.

Actually, the measured SE_T_, SE_A_, and SE_R_ achieved at angle = 0° are always higher than those achieved at angle = 90° regardless of CNF content, as shown in Figure 3h. In addition, MXene/CNF hybrid aerogels (density ≈4 mg cm^–3^) with a higher CNF content exhibit gradually reduced difference in SE values at angles of 0° and 90°, e.g., the SE_T_ and SE_A_ difference values are 26.5 and 21.5 dB, respectively, for the aerogels with 17 wt% CNFs, while those are 4.3 and 2.2 dB, respectively, for the aerogels with 50.0 wt% CNFs. This can be reasonably attributed to the much decreased content of MXene nanosheets when enlarging the CNF fraction, resulting in quickly reduced electrical conductivity and shielding ability as well as weakened internal electric field that is generated in the hybrid aerogels.

We further fabricated hybrid aerogels with various porosities (thus, various densities) by adjusting the water fraction in the MXene/CNF mixed dispersions (Table S2, Supporting Information). Unlike the pure ultralight MXene aerogel (density = 2 mg cm^–3^) that exhibits irregular pore channels and holey cell walls (Figure S14a, Supporting Information), the presence of 17 wt% CNFs enables the formation of ultralight MXene/CNF aerogels (density as low as 1.5 mg cm^–3^) with well‐maintained microstructural characteristics (Figure S14b, Supporting Information). It is worth noting that the fabrication of ultralight aerogel is of critical importance, yet quite challenging, for achieving high EMI shielding performance. The main reason for the ultralight hybrid aerogel is credited to the structural directing effect of the CNFs.^[^ ^25^ ^]^ **Figure** **4**a shows that the EMI SE of 2 mm‐thick pure MXene aerogel (density of 2.0 mg cm^–3^) reaches a SE value of 25.5 dB, which is considerably lower than that of the 2 mm‐thick MXene/CNF hybrid aerogels (SE of 35.5 dB) with an ultralow density of merely 1.5 mg cm^–3^. This indicates the synergistic effects between the 1D CNFs and 2D MXenes for fabricating ultralight aerogels with excellent EMI shielding performance. On the other side, hybrid aerogels with a higher density (up to 20 mg cm^–3^) were also fabricated for widely controlling the EMI SE. For instance, the MXene/CNF aerogel with a density of 8.0 mg cm^–3^ displays a large EMI SE value of 74.6 dB.

**Figure 4:**
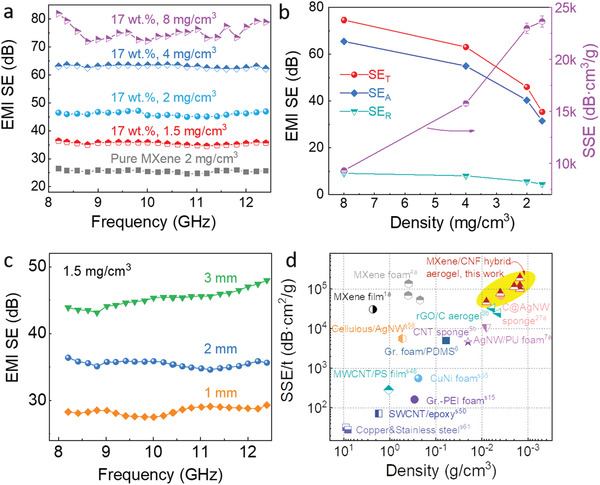
EMI shielding performance of the MXene/CNF hybrid aerogels. a) EMI SE in the X‐band and b) shielding performance and SSE at 10 GHz for the MXene/CNF hybrid aerogels (17 wt% CNF) with varying densities. c) EMI SE in the X‐band at various thicknesses for the MXene/CNF hybrid aerogels (17 wt% CNF) at densities of 1.5 mg cm^–3^. d) Comparison of the MXene/CNF hybrid aerogels' shielding performance with other typical carbon‐, metal‐, and MXene‐based shielding materials: SSE/*t* values of the materials with various densities.

The hybrid aerogels with a higher density typically display larger SE_T_ and SE_A_ values (Figure 4b). Therefore, in the pursuit of ultralight aerogels with excellent EMI shielding performance, one needs to decrease the density while maintaining high SE values. As such, a figure of merit, SSE defined as the ratio of SE to density, is used to evaluate the EMI shielding performance and for comparison with other material systems.^[^ ^5^, ^13^, ^14^ ^]^ A higher SSE represents superior EMI shielding performance achieved in a lightweight material architecture, which is a desired property of next‐generation electronics. Impressively, our MXene/CNF hybrid aerogels have demonstrated quite high and wide‐range SSE values depending on the density of the aerogels. A maximum SSE of 23 633 dB cm^3^ g^–1^ has been achieved in the 2 mm‐thick hybrid aerogel with density of 1.5 mg cm^–3^, surpassing all so far reported shielding materials at similar thickness (Table S3, Supporting Information).

The EMI shielding performance is also thickness dependent; thicker aerogel typically exhibits higher EMI SE values. As showcased in Figure 4c, a 1 mm‐thick MXene/CNF hybrid aerogel (density ≈1.5 mg cm^–3^) displays a SE value of 28.4 dB, which further increases to >45 dB corresponding to a 99.99% attenuation of incident EM waves in the 3 mm‐thick aerogel. Therefore, another figure of merit, SSE/*t* is utilized to evaluate the EMI shielding performance.^[^ ^1^, ^4^, ^9^, ^11^ ^]^ As shown in Figure 4d and Table S3, Supporting Information, our typical MXene/CNF hybrid aerogels exhibit very high SSE/*t* values in a wide range of densities. In particular, superhigh SSE/*t* up to 189 400 dB cm^2^ g^–1^ has been achieved, which is much higher than that of other shielding materials including carbon‐based,^[^ ^3^, ^9^ ^]^ metal‐based,^[^ ^7^, ^27^ ^]^ and other MXene‐based^[^ ^4^, ^17^ ^]^ architectures. The excellent EMI shielding performance of our MXene/CNF hybrid aerogel is attributed to the ultralight nature and the unique oriented biomimetic cell walls that are enabled by the synergistic effects between 1D CNFs and 2D MXene nanosheets.

To sum up, we have demonstrated ultralow‐density and highly flexible MXene/CNF hybrid aerogels with impressive EMI shielding performance. The intrinsic properties and structure of MXene as well as the interactions between CNFs and MXene nanosheets allow the efficient fabrication of highly conductive, robust hybrid aerogels consisting of biomimetic, oriented, intact cell walls. In particular, we have revealed that the EMI SE strongly depends on the angle between the oriented cell walls and the electric field direction of incident EM waves, where maximized SE is achieved as the oriented cell walls are parallel to the electric field direction. Such a novel shielding tuning mode offers a wide range of controllable EMI SE values without altering the frame materials, providing great opportunities for building functional aerogels or devices with oriented strctures for excellent EMI shielding performance. Consequently, EMI SE up to 35.5 and 74.6 dB have been achieved in the 1.5 and 8.0 mg cm^–3^ MXene/CNF hybrid aerogels, respectively. Moreover, the ultralight aerogel showcases impressive SSE and SSE/*t* values, reaching up to 30 660 and 189 400 dB cm^2^ g^–1^, respectively, which have significantly exceeded those of other EMI shielding materials reported so far. The extremely high EMI shielding efficiency from the good coupling among the MXene/CNF hybrid cell walls allows the use of ultralight aerogels for device shielding to help eliminate EM radiation as miniaturization of electronics progresses.

## Conflict of Interest

The authors declare no conflict of interest.

## Supporting information

Supporting Information

## References

[1] a) F. Shahzad , M. Alhabeb , C. B. Hatter , B. Anasori , S. Man Hong , C. M. Koo , Y. Gogotsi , Science 2016, 353, 1137; 27609888 10.1126/science.aag2421

[2] a) H. Zhang , I. Hussain , M. Brust , M. F. Butler , S. P. Rannard , A. I. Cooper , Nat. Mater. 2005, 4, 787; 16184171 10.1038/nmat1487

[3] a) Q. Song , F. Ye , X. Yin , W. Li , H. Li , Y. Liu , K. Li , K. Xie , X. Li , Q. Fu , L. Cheng , L. Zhang , B. Wei , Adv. Mater. 2017, 29, 1701583; 10.1002/adma.201701583 28626927

[4] a) J. Liu , H.‐B. Zhang , R. Sun , Y. Liu , Z. Liu , A. Zhou , Z.‐Z. Yu , Adv. Mater. 2017, 29, 1702367; 10.1002/adma.201702367 28799671

[5] a) D. W. Lu , Z. C. Mo , B. H. Liang , L. L. Yang , Z. F. He , H. Zhu , Z. K. Tang , X. C. Gui , Carbon 2018, 133, 457;

[6] Z. Chen , C. Xu , C. Ma , W. Ren , H. M. Cheng , Adv. Mater. 2013, 25, 1296. 23300002 10.1002/adma.201204196

[7] a) Z. Zeng , M. Chen , Y. Pei , S. I. Seyed Shahabadi , B. Che , P. Wang , X. Lu , ACS Appl. Mater. Interfaces 2017, 9, 32211; 28846376 10.1021/acsami.7b07643

[8] J.‐M. Thomassin , C. Jérôme , T. Pardoen , C. Bailly , I. Huynen , C. Detrembleur , Mater. Sci. Eng., R 2013, 74, 211.

[9] a) Y. Yang , M. C. Gupta , K. L. Dudley , R. W. Lawrence , Nano Lett. 2005, 5, 2131; 16277439 10.1021/nl051375r

[10] X. Shui , D. D. L. Chung , J. Electron. Mater. 1997, 26, 928.

[11] a) B. Wicklein , A. Kocjan , G. Salazar‐Alvarez , F. Carosio , G. Camino , M. Antonietti , L. Bergström , Nat. Nanotechnol. 2014, 10, 277; 25362476 10.1038/nnano.2014.248

[12] Z. Zeng , H. Jin , M. Chen , W. Li , L. Zhou , X. Xue , Z. Zhang , Small 2017, 13, 1701388. 10.1002/smll.201701388 28696564

[13] a) L. Q. Zhang , S. G. Yang , L. Li , B. Yang , H. D. Huang , D. X. Yan , G. J. Zhong , L. Xu , Z. M. Li , ACS Appl. Mater. Interfaces 2018, 10, 40156; 30383958 10.1021/acsami.8b14738

[14] a) C. Zhang , L. McKeon , M. P. Kremer , S.‐H. Park , O. Ronan , A. Seral‐Ascaso , S. Barwich , C. Ó. Coileáin , N. McEvoy , H. C. Nerl , B. Anasori , J. N. Coleman , Y. Gogotsi , V. Nicolosi , Nat. Commun. 2019, 10, 1795; 30996224 10.1038/s41467-019-09398-1 PMC6470171

[15] W.‐T. Cao , F.‐F. Chen , Y.‐J. Zhu , Y.‐G. Zhang , Y.‐Y. Jiang , M.‐G. Ma , F. Chen , ACS Nano 2018, 12, 4583. 29709183 10.1021/acsnano.8b00997

[16] R. Bian , G. He , W. Zhi , S. Xiang , T. Wang , D. Cai , J. Mater. Chem. C 2019, 7, 474.

[17] a) S. Shi , B. Qian , X. Wu , H. Sun , H. Wang , H.‐B. Zhang , Z.‐Z. Yu , T. P. Russell , Angew. Chem., Int. Ed. 2019, 58, 18171; 10.1002/anie.201908402 31591756

[18] a) Y. Ma , Y. Yue , H. Zhang , F. Cheng , W. Zhao , J. Rao , S. Luo , J. Wang , X. Jiang , Z. Liu , N. Liu , Y. Gao , ACS Nano 2018, 12, 3209; 29608277 10.1021/acsnano.7b06909

[19] a) Y. Jiang , X. Xie , Y. Chen , Y. Liu , R. Yang , G. Sui , J. Mater. Chem. C 2018, 6, 8679;

[20] T. A. Schaedler , A. J. Jacobsen , A. Torrents , A. E. Sorensen , J. Lian , J. R. Greer , L. Valdevit , W. B. Carter , Science 2011, 334, 962. 22096194 10.1126/science.1211649

[21] W. Chen , H. Yu , S. Y. Lee , T. Wei , J. Li , Z. Fan , Chem. Soc. Rev. 2018, 47, 2837. 29561005 10.1039/C7CS00790F

[22] a) S. Deville , E. Saiz , R. K. Nalla , A. P. Tomsia , Science 2006, 311, 515; 16439659 10.1126/science.1120937

[23] U. G. Wegst , H. Bai , E. Saiz , A. P. Tomsia , R. O. Ritchie , Nat. Mater. 2015, 14, 23. 25344782 10.1038/nmat4089

[24] C. Zhang , S.‐H. Park , A. Seral‐Ascaso , S. Barwich , N. McEvoy , C. S. Boland , J. N. Coleman , Y. Gogotsi , V. Nicolosi , Nat. Commun. 2019, 10, 849. 30787274 10.1038/s41467-019-08383-y PMC6382913

[25] a) Z. Z. Pan , H. Nishihara , S. Iwamura , T. Sekiguchi , A. Sato , A. Isogai , F. Kang , T. Kyotani , Q. H. Yang , ACS Nano 2016, 10, 10689; 27809476 10.1021/acsnano.6b05808

[26] N. Yousefi , X. Sun , X. Lin , X. Shen , J. Jia , B. Zhang , B. Tang , M. Chan , J. K. Kim , Adv. Mater. 2014, 26, 5480. 24715671 10.1002/adma.201305293

[27] a) Y.‐J. Wan , P.‐L. Zhu , S.‐H. Yu , R. Sun , C.‐P. Wong , W.‐H. Liao , Small 2018, 14, 1800534; 10.1002/smll.201800534 29847702

