# Supplementary material for: Nanocellulose‐MXene Biomimetic Aerogels with Orientation‐Tunable Electromagnetic Interference Shielding Performance
Source: Adv Sci (Weinh). 2020 Jun 28;7(15):2000979. doi: 10.1002/advs.202000979 (PMC7404164; doi:10.1002/advs.202000979)
Supplement: Supplementary file 1 — Supporting Information [file ADVS-7-2000979-s001.pdf]

© 2020 Wiley-VCH GmbH

ADVANCED SCIENCE
Open Access

Supporting Information

for *Adv. Sci.*, DOI: 10.1002/advs.202000979

Nanocellulose-MXene Biomimetic Aerogels with Orientation-Tunable Electromagnetic Interference Shielding Performance

Zhihui Zeng, Changxian Wang, Gilberto Siqueira, Daxin Han, Anja Huch, Sina Abdolhosseinzadeh, Jakob Heier, Frank Nüesch, Chuanfang (John) Zhang, Gustav Nyström**

Supporting Information

Nanocellulose-MXene Biomimetic Aerogels with Orientation-Tunable Electromagnetic Interference Shielding Performance

Zhihui Zeng, Changxian Wang, Gilberto Siqueira, Daxin Han, Anja Huch, Sina Abdolhosseinzadeh, Jakob Heier, Frank Nüesch, Chuanfang (John) Zhang*, Gustav Nyström*

*Email: Gustav.Nystroem@empa.ch; Chuanfang.Zhang@empa.ch

Experimental Section

Fabrication of large lateral size of MXene with majority of single layer. Multilayered MXene was produced through the MILD method based on lithium fluoride (LiF, Sigma Aldrich, USA) and hydrochloric acid (HCl, 37%, Sigma Aldrich, USA) mixture. Prior to the etching, the mixture was prepared by adding 1 g of LiF to 20 mL 9M HCl under stirring till LiF completely dissolved and formed a clear solution. Then, 1 g Ti_3AlC_2 MAX was slowly added to that solution under vigorous stirring. After reacting at room temperature for 24 h, the mixture was centrifuged at 3500 rpm for 5 min. The supernatant was decanted and 40 mL of deionized water was added to re-disperse the sediments. Then the suspension was subjected to another centrifugation at 3500 rpm for 5 min. This decanting-re-dispersing-centrifuging process was repeated for 6 times until the pH of the supernatant reached around six, when the multilayered MXene starts to delaminate. Finally, the suspension was vigorously shaken for 20 min, and subjected to centrifugation for 30 min at 3500 rpm. The supernatant was collected and labelled as d-$Ti_3C_2T_x$ MXene.

Fabrication of the CNFs. First, an oxidation reaction of the Elemental Chlorine Free (ECF) fibers (extracted from bleached softwood pulp fibers, Mercer Stendal Company, Berlin, Germany) was performed. Briefly, the cellulose fibers were dispersed in water to obtain a 2 wt% suspension. Then the fiber suspension was mixed with TEMPO and sodium bromide (NaBr) aqueous solution at concentrations of 0.1 and 1.0 mmol per gram of cellulose pulp, respectively. The pH was adjusted to 10 with sodium hydroxide solution (1 mol/L). A

concentration of 10 mmol sodium hypochlorite was employed per gram of cellulose pulp. The TEMPO-oxidized cellulose fibers were thoroughly washed until the conductivity was similar to that of distilled water. Next, the oxidized and purified cellulose fibers were dispersed in water to a concentration of 2% (w/w) and ground using a Supermass Colloider (MKZA10-20J CE Masuko Sangyo, Japan) to obtain a CNF dispersion. Finally, TEMPO-oxidized CNFs were further processed and disintegrated using a high-shear homogenizer (M-110EH, Microfluidics Ind., Newton, MA - USA) with two interaction chambers with Z-shape design 200 and 100 μm in diameter and pressures up to eight bar to improve the degree of fibrillation of the CNFs.

Fabrication of the MXene/CNF hybrid aerogels with oriented cell walls. MXene/CNF mixed dispersion were first prepared by simple magnetic stirring for 2 h of as-prepared MXene and CNF dispersions. The MXene/CNF hybrid aerogels were assembled by freezing the MXene/CNF mixed dispersion in a Teflon mold attached to a metal base (cold finger) with liquid nitrogen after the water fraction is regulated, following by drying the prepared hydrogels in the freeze-drying vessel (–80 °C and 4 Pa). Herein, a large temperature gradient from the bottom to the top in the dispersion resulted in the unidirectional growth of the lamellar ice crystals. The ice crystals excluded the MXene/CNF on the surface to form the MXene/CNF hybrid cell walls and led to final aerogels with unidirectional pore channels/cell walls after the ice crystals were sublimated in the freeze-drying process. The anisotropic honeycomb-like samples can be cut effortlessly to obtain various angles of the pore channel/cell walls for the EMI shielding test. Various shapes of porous architectures could be obtained by selecting various molds. The mass ratio of the MXene to CNF was controlled in the dispersion to prepare the MXene/CNF hybrid aerogels with various CNF contents. The water fraction (or concentration) of the mixed dispersion was controlled to obtain the hybrid aerogels with various densities. For instances, the mass ratio of MXene to CNF is controlled to 5 to 1 in the suspension with a total concentration of 0.29 wt%, leading to a MXene/CNF

hybrid aerogels with 17 wt% CNF and density of 4 mg/cm^3. The identical freeze-drying process of MXene dispersion led to the preparation of pure MXene aerogels without any CNF content.

***Characterization*:** The microstructure is characterized by Scanning Electron Microscopy (SEM, FEI NanoSEM 230), Atomic Force Microscopy (AFM, Bruker ICON3), and Transmission Electron Microscopy (bright field imaging TEM with 200 kV using JEOL 2200 FS). For the TEM characterization, copper grids (200 mesh) with formvar/carbon film were first pretreated in the plasma cleaner for 10 seconds, to make them more hydrophilic. The plasma-cleaned grids were placed face down on a drop of CNF or MXene suspension for 60 seconds and the excess of the suspension was wicked off using filter paper. The sample was then stained by placing the grid face down on a drop of uranyl acetate (2% solution in water) again for 60 seconds. The excess solution was blotted using filter paper and the grid dried at room temperature for at least 24 h prior to imaging. In addition, aerogel samples were embedded in a low-viscosity epoxy resin and the castings are polymerized at 70°C in 24 hours. Sections were prepared with a Reichert-Jung Ultracut using a diamond knife. Ultrathin cross-sections of embedded aerogel samples with a thickness of approximately 70 nm were cut and placed on a formvar/carbon coated copper grid (200 mesh) for cross-section characterization. The XRD patterns were obtained in specular reflection mode (Cu K-α radiation, PANalytical X'Pert) at room temperature The functional groups of the aerogels were characterized by FTIR (Spectrum 100 from Perkin Elmer) spectroscopy. The four-probe method with a Keithley 2400 sourcemeter was used to test the resistance of the samples at room temperature. The resistance is employed to calculate the electrical conductivity (σ) by the equation $\sigma = L / (R{\cdot}w{\cdot}t)$, where L, w, and t were the length between measuring electrodes, the width, and the thickness of the samples, respectively. The measured electrical conductivities of the aerogels with different directions were similar due to the interconnected conductive cell walls. The compression behavior of the porous sample was evaluated using a dynamic mechanical

analyzer (DMA, TA Q800) with a DMA strain rate mode. At least five duplicates for each type of aerogels were tested. Unless explicitly mentioned, the aerogels were compressed in the longitudinal direction, *i.e.*, the direction parallel to the aligned cell walls, in order to describe the cell walls' microstructure better. The waveguide method using a vector network analyzer (Agilent 8517A) is employed to measure EMI SE of the samples with size of 22.86 mm × 10.16 mm (length × width) in the frequency range of 8.2–12.4 GHz (X-band). More than five specimens were tested for each type of aerogel. The S-parameters of each sample were recorded and employed to calculate the SE_T, SE_R, and SE_A. It is worth noting that the sample thickness could be adjusted but it could not be larger than 9.46 mm because the waveguide chamber's dimensions were 22.86 mm (length) × 10.16 mm (width) × 9.46 mm (height). The electric field direction was in the width direction and the propagation direction of tested wave was in the thickness direction of the waveguide chamber. Unless specially mentioned, in the case of EMI SE measurement, the propagation direction of incident wave was normal/vertical to the longitudinal plane of the honeycomb-like aerogels to obtain a most utilization of the cell wall-void interfaces. At this point, the pore channels'/cell walls' oriented direction can be tuned to various angles with the electric field direction (width direction of tested sample) to realize the influences of the angles on the EMI shielding performance for the aerogel samples with a fixed thickness (wave propagation direction). Therefore, for general EMI shielding comparison to clarify the influence of the CNF content, this angle between oriented cell walls and electric field direction of incident EM waves is also fixed at zero unless specially mentioned.

Finite element analysis of cell walls'/pore channels' orientation induced tunable EMI shielding performance for the honeycomb-like porous architectures. Finite element analysis (FEA) for simulating the EMI shielding efficiency was implemented by using COMSOL Multiphysics, a finite-element analysis and solver software. The simulations were performed in the EM waves' frequency domain module with a 2D hexagonal honeycomb model. The

width and thickness of the cell wall, derived from SEM and TEM images, were set to 10 um (corresponding to a pore size of 20 um) and 36 nm, respectively. The gross thickness of the whole structure is 2 mm with 116 honeycomb unit cells periodically arranged in zigzag direction. Two ports (port 1 and port 2) were established in the air domain to calculate the S-parameters. The port 1 was activated with 1 W input power in electric field mode. Perfectly matched conditions were imposed on the air domain to eliminate interference from the reflected waves. The largest mesh element size was set lower than 1/10th of the shortest incident wavelength to ensure the accuracy.

The reflection coefficient (R), transmission coefficient (T), and absorption coefficient (A) were calculated by S parameters (S_{11} and S_{21}) according to the following equations

$$R = |S_{11}|^2$$

$$T = |S_{21}|^2$$

$$A = 1 - R - T$$

Finally, SE_A, SE_R, and SE_T are obtained by using following equations:

$$SE_R = 10\log(\frac{1}{1-R})$$

$$SE_T = 10\log(\frac{1}{T})$$

$$SE_A = SE_T - SE_R$$

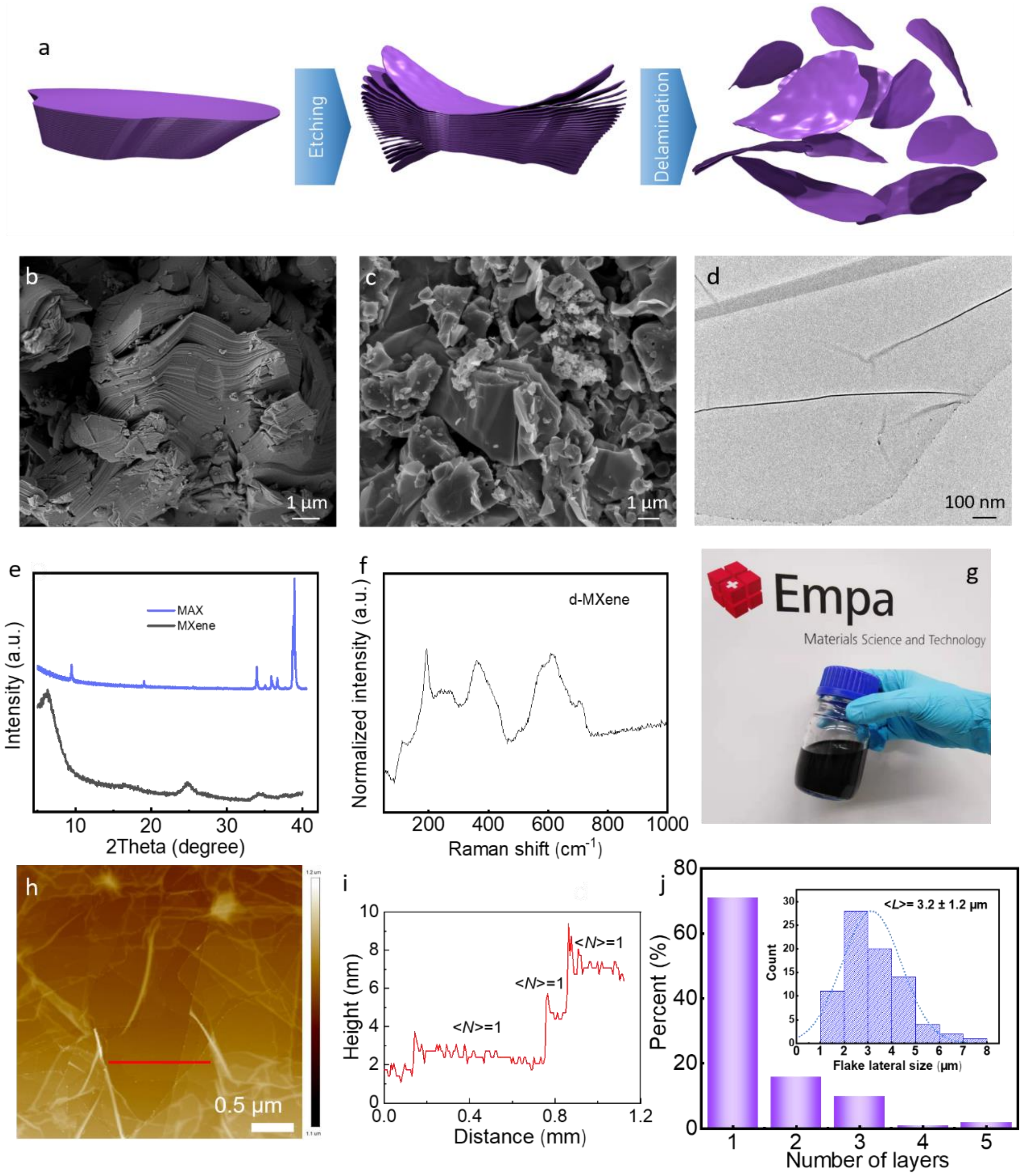

Figure S1. **Preparation and properties of the as-prepared MXene layers.** (a) Schematic of the preparation process of the MXene. SEM images of (b) MAX phase and (c) multilayered MXene, and (d) TEM Image of delaminated MXene (d-MXene). (e) XRD patterns of MAX phase, multilayered MXene, and delaminated MXene (d-MXene), (f) Raman spectrum of the d-MXene, (g) optical image of the d-MXene aqueous dispersion. (h) typical AFM images of the d-MXene layers of the d-MXene and (i) the height profiles of the line in image H. (j) Dimensional statistics of the d-MXene, showing a majority of monolayer and a large lateral size (the total number of samples was 100).

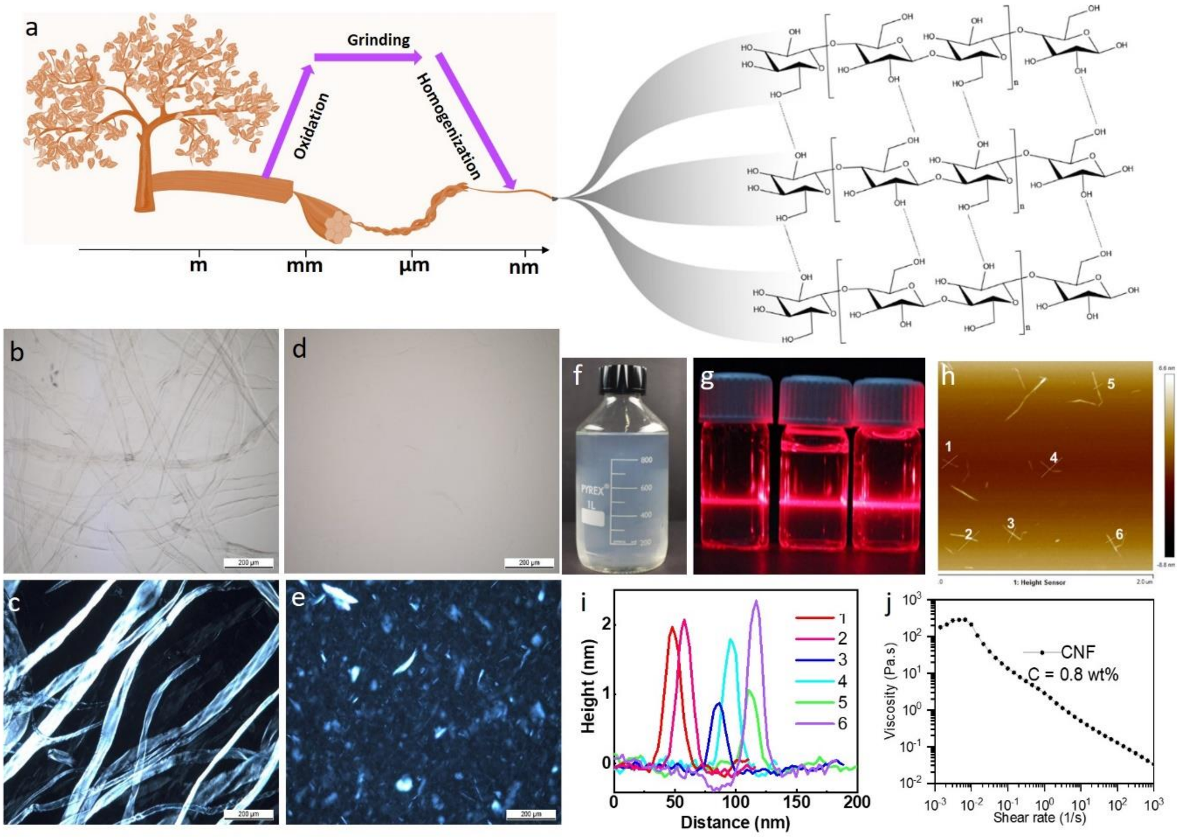

Figure S2. **Properties of the CNFs.** (a) Schematic of CNF preparation process, method, and chemical structure of the CNF. Optical microscopy images of the CNF dispersions (b, c) before and (d, e) after grinding treatment, showing a high degree of fibrillation of the CNFs after the treatment. In image D, the ultrathin CNFs cannot be observed in this mode. (f) The optical images of the as-prepared CNF dispersion and the (g) typical Tyndall effect of the CNF suspension with various concentrations of 1.2 wt%, 0.5 wt% and 0.1 wt% from left to right. (h) AFM images of the CNFs and (i) the height profiles of the lines in image H, showing the thin dimension and large aspect ratio of CNFs. (j) The viscosity of 0.8 wt% CNF dispersion (low viscosity is shown with a value of 0.16 Pa·s at a speed of 46.4 s^{-1}).

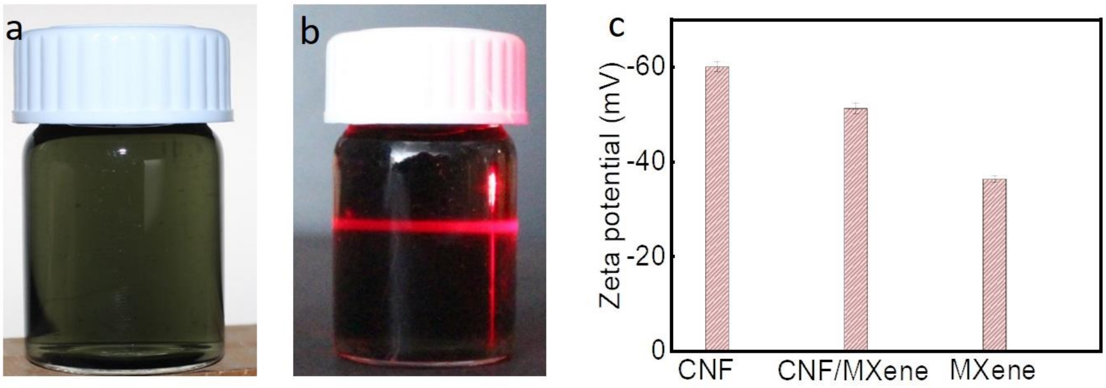

Figure S3. Properties of the MXene/CNF hybrid dispersion. (a) Optical images of the MXene/CNF mixed dispersion and (b) the corresponding Tyndall effect. (c) The zeta potentials of the CNF, CNF/MXene mixed, and MXene suspension. Similar hydrophilicity and negative charges of MXene and CNF make their aqueous suspension stable, which can be demonstrated by the typical Tyndall effect. This is instrumental in constructing the 3D hybrid aerogels.

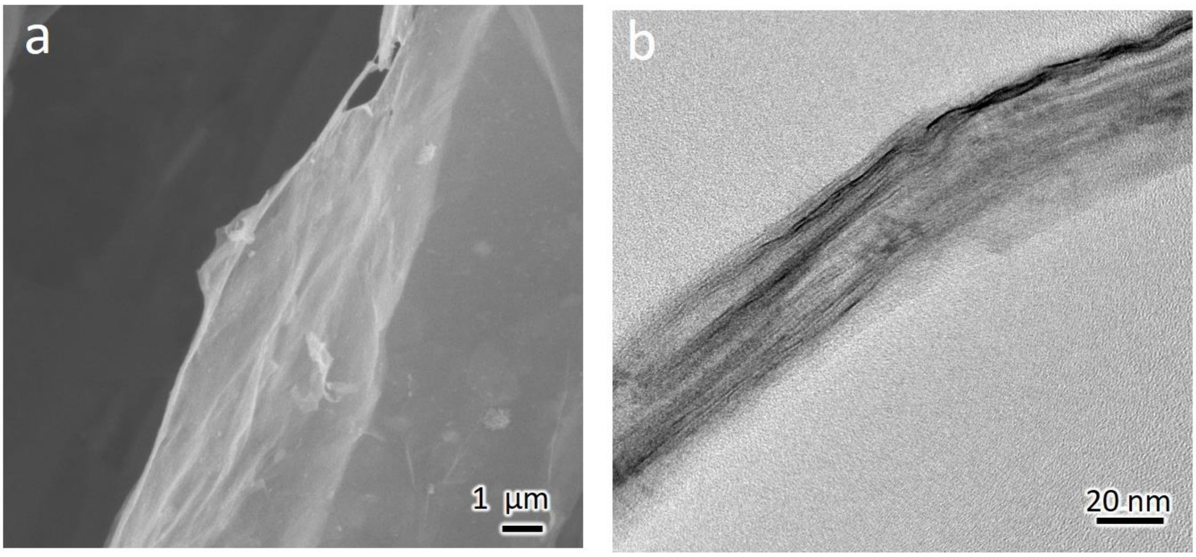

Figure S4. Microstructure of the cell walls. (a) SEM image and (b) cross-sectional TEM image of the hybrid cell walls in the MXene/CNF hybrid aerogels with 17 wt% CNF.

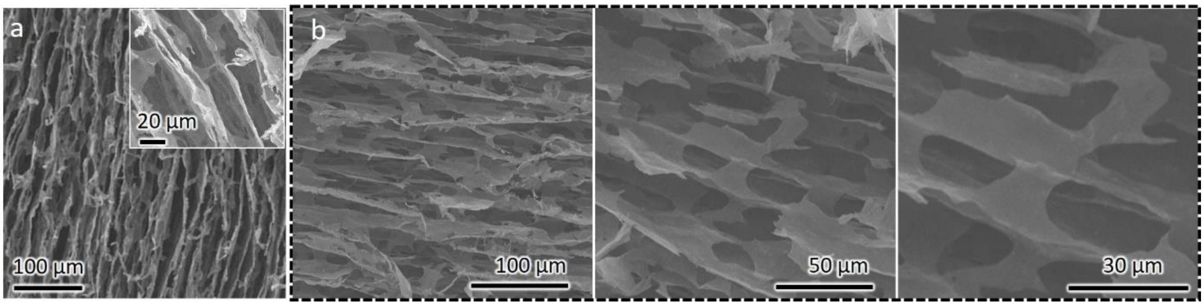

Figure S5. SEM images of the MXene-based aerogels. Typical SEM images of the (a) MXene/CNF hybrid aerogels with 50 wt% CNFs, and (b) pure MXene aerogels without CNF, at a density of 4 mg/cm^3. Most cell walls are holey due to the low gelation capability of the MXene layers in the pure MXene aerogels.

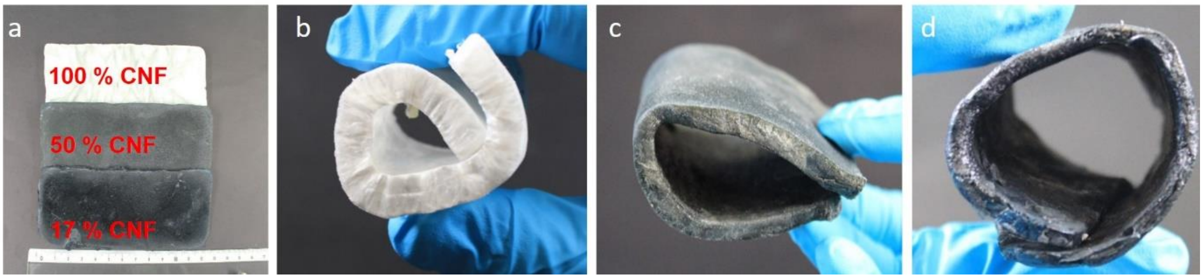

Figure S6. **Optical images of the MXene-based aerogels.** (a) Large-area (12 cm × 6 cm) CNF aerogels (top), MXene/CNF hybrid aerogels with 50 wt% CNF (middle), and MXene/CNF hybrid aerogels with 17 wt% CNF (bottom), at densities of 4 mg/cm^3, flexible performance of (b) CNF aerogels, and MXene/CNF hybrid aerogels with (d) 50 wt% CNF and (d) 17 wt% CNF.

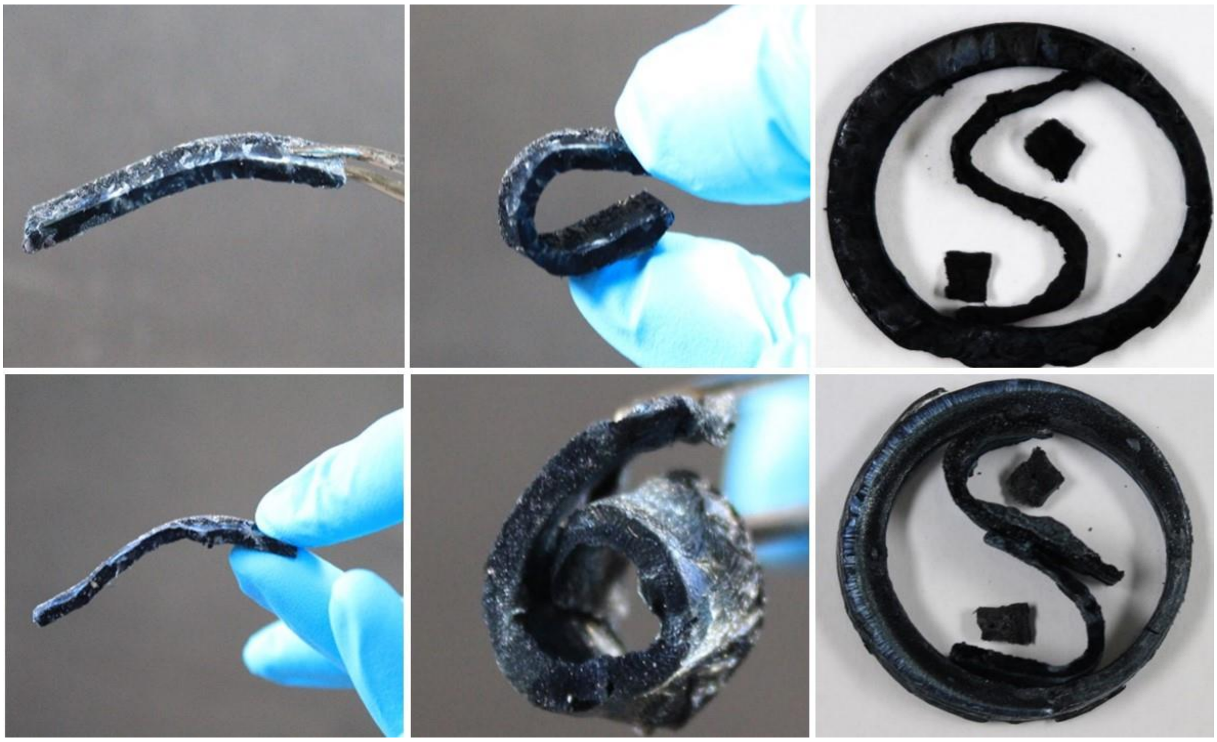

Figure S7. **Flexible performance of the MXene-based aerogels**. Optical images demonstrating the flexible performance including flexibility and bendability of the easy-shaped MXene/CNF hybrid aerogels with 17 wt% CNF at a density of 4 mg/cm^3.

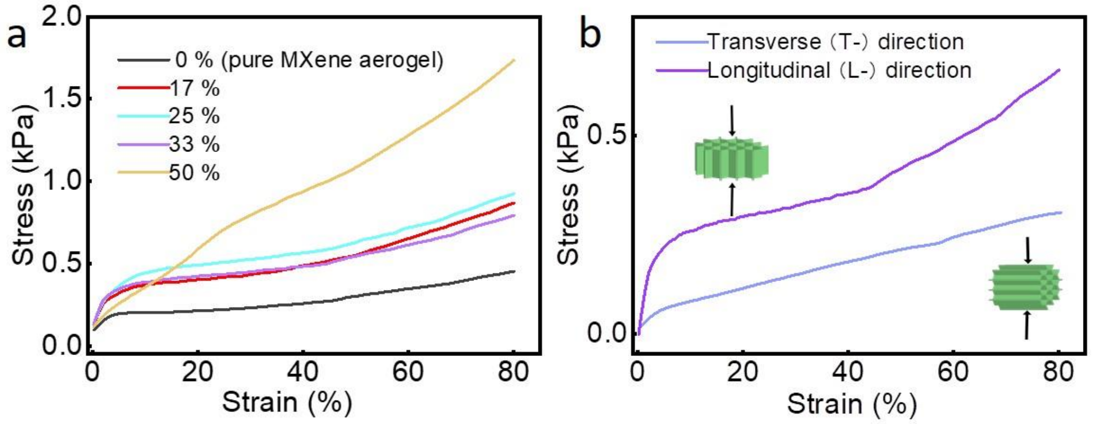

Figure S8. **Mechanical Properties of the MXene-based aerogels.** Compressive curves of the MXene/CNF hybrid aerogels (a) with various CNF contents in longitudinal direction, and (b) with 33 wt% CNFs in longitudinal (L-) and transverse (T-) directions.

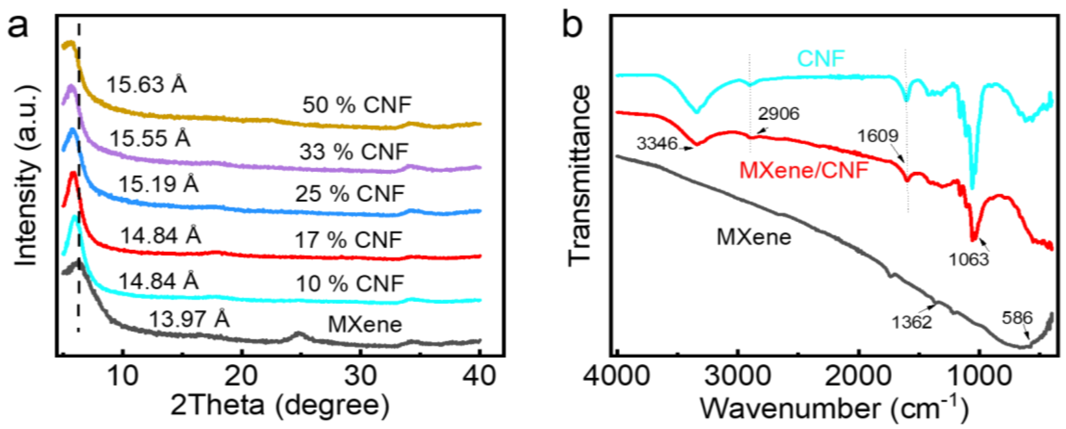

Figure S9. (a) XRD patterns of the MXene-based aerogels (4 mg/cm^3) with various CNF contents. (b) FTIR spectra of typical CNF, MXene and MXene/CNF aeroegls. MXene has two typical peaks at 1362 and 586 cm^{-1}, corresponding to the surface terminal group of C–F and −OH, respectively. After being mixed with CNFs, the cellulose characteristic absorption bands at 2920 (C–H stretching) and 1639 cm^{-1} (−OH bending) are observed in the FTIR spectrum of the hybrid aerogels.

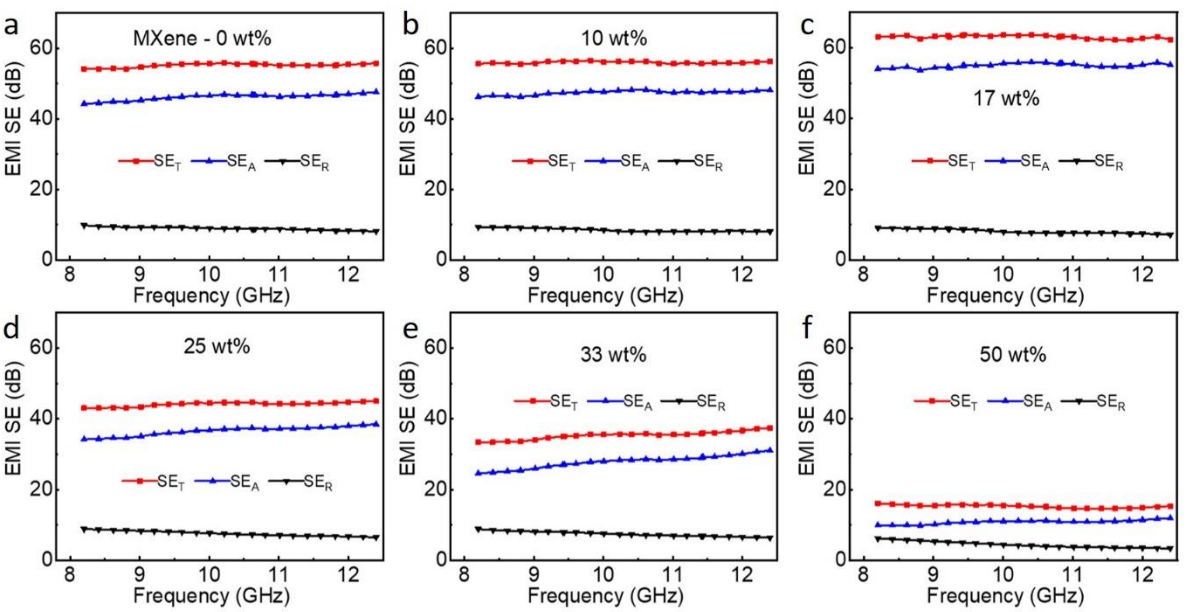

Figure 10. EMI shielding performance of the MXene/CNF hybrid aerogels with various CNF contents from 0-50 wt%, at a density of 4 mg/cm^3.

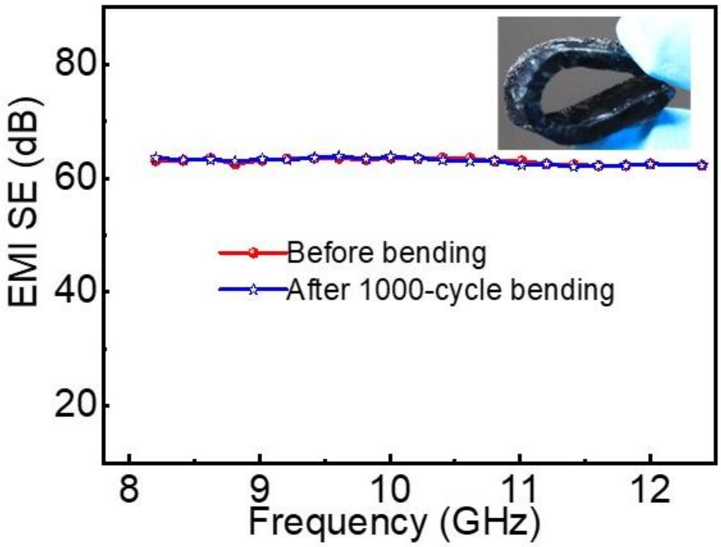

Figure S11. EMI shielding performance of the MXene/CNF hybrid aerogels with 17 wt% CNF before and after 1000-cycle bending.

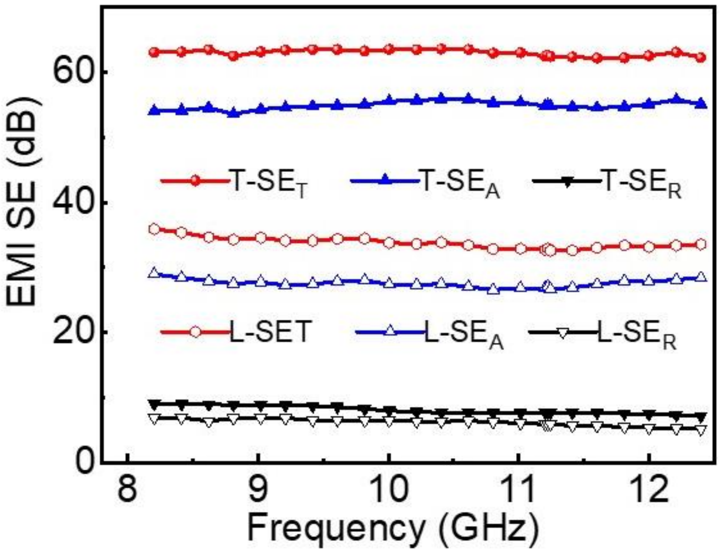

Figure S12. EMI shielding performance of the MXene/CNF hybrid aerogels with 17 wt% CNF at a density of 4 mg/cm^3 in the transverse (T-SE_T, T-SE_A, and T-SE_R) and the longitudinal (L-SE_T, L-SE_A, and L-SE_R) directions. The difference of the EMI SE is attributed to the different propagation direction of the incident EM waves that meet and interact with different void-cell walls interfaces, leading to a different multi-reflection.

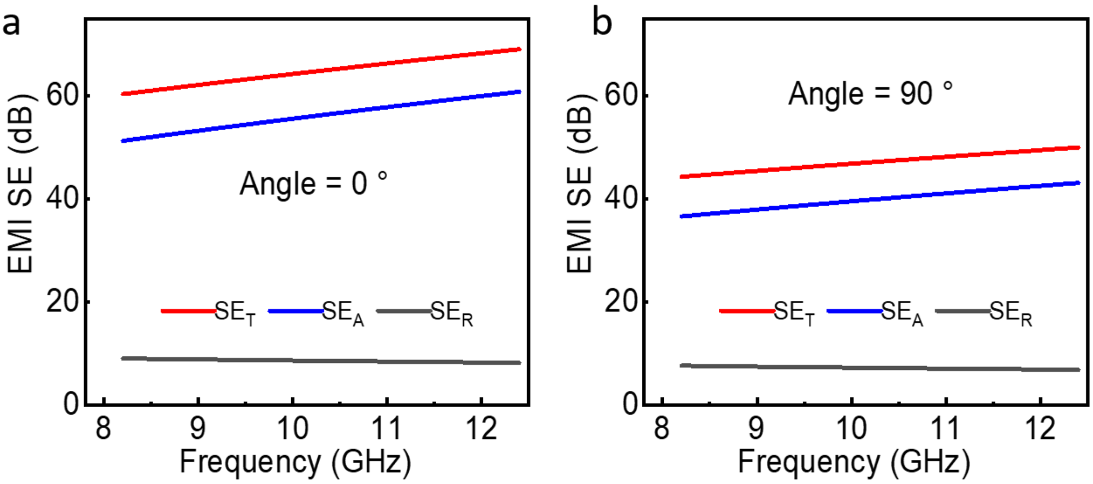

Figure S13. Theoretical simulation and calculation. Finite element analysis of EMI shielding performance of the honeycomb-like porous architectures in different angles between pore channels'/cell walls' orientation and electric field direction of incident EM waves: (a) 0° and (b) 90°.

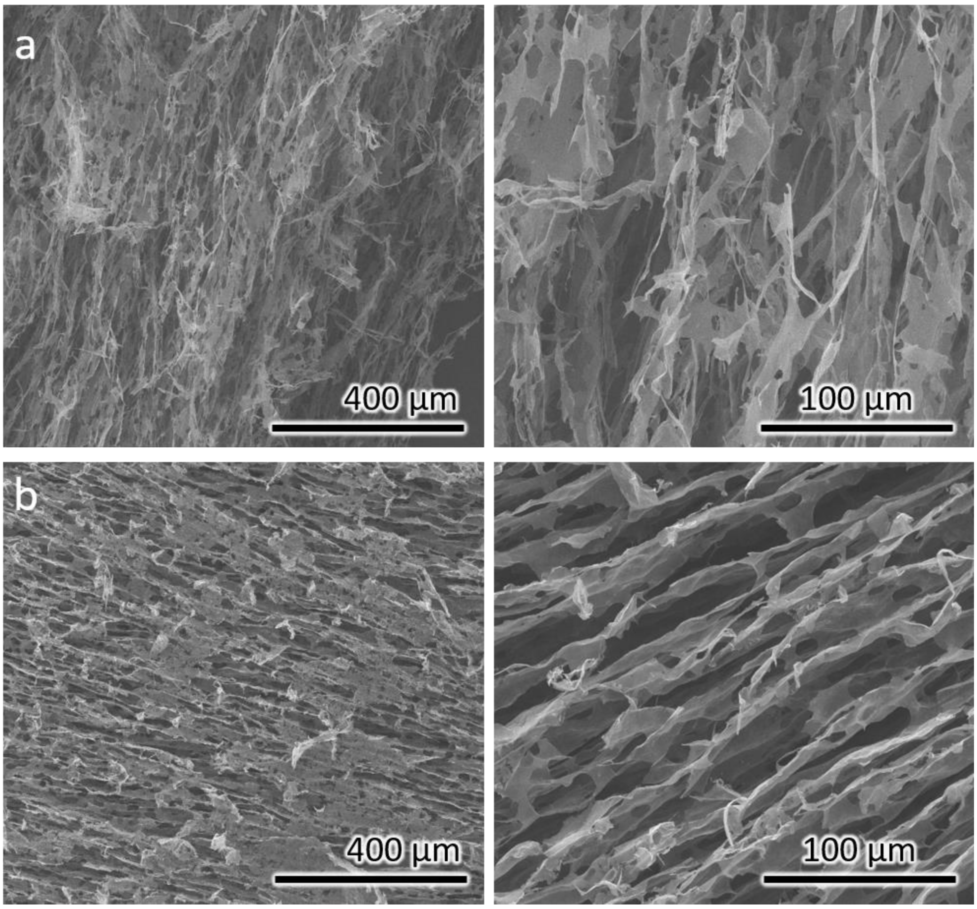

Figure S14. Microstructure of the MXene-based aerogels. SEM images of (a) the pure MXene aerogels at a density of 2 mg/cm^3, and (b) the MXene/CNF hybrid aerogels with 17 wt% CNF at a density of 1.5 mg/cm^3.

Table S1. **Properties of the MXene/CNF hybrid aerogels with various CNF contents at a similar density of 4 mg/cm^3.**

CNF mass ratio (wt%)	2θ (°)	d-spacing (Å)	MXene content (vol%)	CNF content (vol%)	Porosity (%)	Conductivity (S/m)
0	6.32	13.97	0.17	0	~99.83	29.3
10	5.95	14.84	0.153	0.027	~99.82	28.6
17	5.95	14.84	0.135	0.045	~99.82	27.6
25	5.81	15.19	0.117	0.063	~99.82	19.6
33	5.68	15.55	0.100	0.080	~99.82	6.46
50	5.65	15.63	0.068	0.112	~99.82	0.35

Table S2. Properties of 17 wt% CNF/MXene hybrid aerogels with various densities at a thickness of 2 mm.

Density (mg/cm^3)	MXene content (vol%)	Porosity	Weight reduction (%)	Conductivity (S/m)	EMI SE (dB)	SSE (dB·cm^3/g)
20	1.00	0.991	99.1	/	Over range	/
8	0.274	0.996	99.6	108.7	74.56±1.23	9320
4	0.135	0.998	99.8	27.6	63.05±1.02	15763
2	0.069	0.999	99.9	1.44	45.99±0.99	22995
1.5	0.051	0.9993	99.9	1.17	35.35/	23633

/: Not measured or not calculated

Note S1. Calculation of porosity of the porous architectures

The porosity of the aerogels is defined as $1 - \rho / \rho_0$, where ρ and ρ_0 are the density of the porous architectures and relevant solid films, respectively. The densities of the MXene/CNF hybrid aerogels are obtained by the weighing method and combined with the density of relevant MXene/CNF hybrid films (around 2.2 g/cm^3). From here the porosity of the porous scaffolds can be calculated.

Table S3. EMI shielding performance of various shielding materials.

Materials	EMI SE (dB)	Density (mg/cm^3)	Thickness (mm)	SSE (dB·cm^3/g)	SSE/*t* (dB·cm^2/g)
**MXene-based porous and solid shields**					
MXene/CNF hybrid aerogels (this work)	74.56	8.0	2.0	9320	46600
	63.05	4.0	2.0	15763	78813
	45.99	1.5	3.0	30660	102200
	35.45	1.5	2.0	23633	118167
	28.41	1.5	1.0	18940	189400
Mono-layered MXene aerogel (this work)	55.2	4.0	2	13800	69000
	25.5	2.0	2	12750	63750
MXene foam[1]	32	390	0.006	82	137000
	70	220	0.06	318	53030
Few-layered MXene aerogel[2]	61.2	6.26	2	9904	49520
MXene/CNT aerogel[3]	104	42	3	2476	8254
	62.8	25	1	2511	25108
MXene-POSS-NH_2 aerogel[4]	34.5	/	2	/	/
MXene/PVA aerogel [5]	28	10.8	5	2586	5136
MXene/SA film[6]	57	~2317	0.008	24.6	30830
MXene film[6]	68	~2394	0.011	28.4	25863
MXene/Nanocellulose film [7]	24	2000	0.047	12	2647
	25	1136	0.0167	22	1326
MXene@PS solids [8]	62	1051	2	59.0	29.5
MXene/RGO-epoxy solids[9]	56.4	/	2	/	/
**Carbon-based porous and solid shields**					
CNF/PS foam[10]	19		/	/	/
CNT/PS foam[11]	19	574	/	33.1	/
Graphene/PVDF foam[12]	28		/	/	/
Graphene/PMMA foam[13]	19	792	2.4	24	100
Graphene/PS foam[14]	29	450	2.5	64.4	258
Graphene /PEI foam[15]	9-12.8	~290	2.3	31– 44	135–192
Graphene@Fe_3O_4/PEI foam[16]	15-18	400	2.5	37.5–44	150–176
CF/PP foam[17]	25	735	3.1	34	109
Stainless-steel fiber/PP foam[18]	48	640	3.1	75	242
MWCNT/PLA foam[19]	23	299	2.5	77	308
MWCNT/PVDF foam[20]	57	750	2	76	380
MWCNT/WPU foam[21]	23.0	20	2.3	1148	4991
	21.1	39	1	541	5410
MWCNT/cellulose aerogel[22]	20-35	~37-47	2.5	425-944	1700-3776
Cellulose aerogel coated with MWCNT[22]	35-40	~69-75	2.5	466-519	1864-2078
Graphene foam based PDMS foam[23]	30	60	1	~500	~5000
Graphene foam/CNT/PDMS[24]	75	90	2	833	4165
Graphene-coated PU foam[25]	19.9	30.0	20	663.3	3320
Graphene foam coated with PEDOT:PSS[26]	69.1	22.1	1.5	3124	20837
Graphene based composite aerogel[27]	37	70	3	529	1762
Sponged-supported RGO aerogel[28]	24	16.7	12	1437	1198

CNT/multi-layered graphene foam[29]	~38	5.8	1.6	6600	~40000
Graphene/cellulose-derived carbon foam[30]	47.8	2.8	5.0	16890	33780
Graphene/lignin-derived carbon aerogels [31]	23.2	2.5	2	9280	46400
	14.3	2.5	1	5720	57200
Graphene aerogel[31]	22.3	4.5	2	4956	24778
Carbon foam-CNT/carbon fiber foam[32]	21	12.4	5.0	1690	3370
CNT mat[33]	30	/	0.001	/	/
CF mat[34]	23	/	0.06	/	/
Ni/CF mat[34]	29	/	0.06	/	/
Fe_3O_4/CNF mat[35]	68	/	0.7	/	/
CNF mat[36]	81.1	219	4.6	370	804.3
	52.2	134	2.9	390	1361.6
Graphene/CNA[37]	58.4		2.0	/	/
Carbon/Graphene foam[38]	24	721	0.024	33.3	13889
Graphene foam[39]	25.2	60	0.3	420.0	14000
Phthalonitrile-based carbon foam[40]	51.2	150	2	341.1	1707
Commercial carbon foam[41]	40	166	2	241	1250
CNT sponge[42]	22	20	2.38	1100	4622
MWCNT/PTT[43]	22	/	2		/
MWCNT/PP [44]	24	899	2.8	~26.7	95
	35	/	1.0	/	/
CNF sponge/Epoxy[45]	40	/	2	/	/
MWCNT/ABS[46]	50	1050	1.1	~47.6	433
Carbon black (CB)/ABS[46]	22	/	1.1	~20.9	~190
Carbon nanofiber (CNF)/ABS[46]	35	/	1.1	/	/
MWCNT/PC[47]	25	/	1.85	/	
MWCNT/PS[48]	60	/	2	~57	285
MWCNT/WPU[49]	24-50	1200	0.05-0.32	20-42	3408
SWCNT/epoxy[50]	25	1748	2	~14.3	72
SWCNT/PU[51]	18	/	2	~17	80
Graphene/WPU[52]	32		2	~30.5	153
CB/EPDM[53]	18	/	5.5	/	
Flexible Graphite [54]	110	1100	0.2	100	500
**Metal-based porous and solid shields**					
CuNi foam[55]	15-25	~240	1.5	63–104	420–690
CuNi-CNT foam[55]	40-54.6	~230	1.5	174–237	116–1580

Porous cellulose papers coated with Ag NWs[56]	48.6	530	0.164	91.7	5584
Ag NWs/PI foam[57]	17-23.5	22	5	1068-772	2136 -1544
Ag NWs/WPU foam[58]	20.0-64.0	8.0	2.3	2500-1422	10970-6184
Ag NW@C hybrid sponge [59]	37.9	3.8	1	9921	99214
	70.1	3.8	3	18350	61169
Cu NWs aerogels[60]	~17		9.46	/	/
Cu NW@ graphene aerogels[60]	52.5	166	9.46	3170	3921.8
Copper[61]	90	8960	3.1	10	32
Nickel[61]	82	8900	/	9.2	
Stainless steel[61]	89	8100	4	11	28
(2 µm) Ni fibers/PES[61]	58	1871	2.85	31	109
(20 µm) Ni fibers/PES[61]	4	250	2.85	16	
Ni filaments/PES[60]	~87	/	2.85	47	165
Aluminium flakes/PES[62]	35-39	/	2.92	/	/
Ag NW/PANI[63]	48	1250	0.0133	38.4	28872
Ag NW/epoxy[64]	25.09	1255	0.040	20.0	5018
Ag NP/epoxy[64]	5.06	1234	0.040	4.1	1012
Ag NW/PVA[64]	30.1	1123	0.040	26.8	6691
Ag NW/PS[65]	31.85	1051	0.8	30.3	379
Cu NW/PS[66]	35	1051	0.21	33.3	158.7
Al foil[6]	66	2700	0.008	24.4	30555
Cu foil[6]	70	8960	0.010	7.8	7812

/: unclear or uncalculated value; the numbers in the square brackets denote the numbers of references which are at the end of the supporting information.

[1] J. Liu, H.-B. Zhang, R. Sun, Y. Liu, Z. Liu, A. Zhou, Z.-Z. Yu, *Adv. Mater.* **2017**, 29, 1702367.

[2] R. Bian, G. He, W. Zhi, S. Xiang, T. Wang, D. Cai, *J. Mater. Chem. C* **2019**, *7*, 474.

[3] P. Sambyal, A. Iqbal, J. Hong, H. Kim, M. K. Kim, S. M. Hong, M. Han, Y. Gogotsi, C. M. Koo, *ACS Appl.Mater. Interfaces* **2019**, *11*, 38046.

[4] S. Shi, B. Qian, X. Wu, H. Sun, H. Wang, H.-B. Zhang, Z.-Z. Yu, T. P. Russell, *Angew. Chem. Int. Ed.* **2019**, doi.org/10.1002/anie.201908402.

[5] H. Xu, X. Yin, X. Li, M. Li, S. Liang, L. Zhang, L. Cheng, *ACS Appl.Mater. Interfaces* **2019**, *11*, 10198.

[6] F. Shahzad, M. Alhabeb, C. B. Hatter, B. Anasori, S. Man Hong, C. M. Koo, Y. Gogotsi, *Science* **2016**, 353, 1137.

[7] W.-T. Cao, F.-F. Chen, Y.-J. Zhu, Y.-G. Zhang, Y.-Y. Jiang, M.-G. Ma, F. Chen, *ACS Nano* **2018**, 12, 4583.

[8] R. Sun, H.-B. Zhang, J. Liu, X. Xie, R. Yang, Y. Li, S. Hong, Z.-Z. Yu, *Adv. Funct. Mater.* **2017**, 27, 1702807.

[9] S. Zhao, H.-B. Zhang, J.-Q. Luo, Q.-W. Wang, B. Xu, S. Hong, Z.-Z. Yu, *ACS Nano* **2018**, 12, 11193.

[10] Y. Yang, M. C. Gupta, K. L. Dudley, R. W. Lawrence, *Adv. Mater.* **2005**, *17*, 1999.

[11] Y. Yang, M. C. Gupta, K. L. Dudley, R. W. Lawrence, *Nano Lett.* **2005**, *5*, 2131.

[12] V. Eswaraiah, V. Sankaranarayanan, S. Ramaprabhu, *Macrom. Mater. Engineer.* **2011**, *296*, 894.

[13] H. B. Zhang, Q. Yan, W. G. Zheng, Z. He, Z. Z. Yu, *ACS Appl. Mater. Interf.* **2011**, *3*, 918.

[14] D. X. Yan, P. G. Ren, H. Pang, Q. Fu, M. B. Yang, Z. M. Li, *J. Mater. Chem.* **2012**, *22*, 18772.

[15] J. Ling, W. Zhai, W. Feng, B. Shen, J. Zhang, W. Zheng, *ACS Appl. Mater. Interf.* **2013**, *5*, 2677.

[16] B. Shen, W. Zhai, M. Tao, J. Ling, W. Zheng, *ACS Appl. Mater. Interf.* **2013**, *5*, 11383.

[17] A. Ameli, P. U. Jung, C. B. Park, *Carbon* **2013**, *60*, 379.

[18] A. Ameli, M. Nofar, S. Wang, C. B. Park, *ACS Appl. Mater. Interf.* **2014**, 6, 11091.

[19] T. Kuang, L. Chang, F. Chen, Y. Sheng, D. Fu, X. Peng, *Carbon* **2016**, 105, 305.

[20] H. Wang, K. Zheng, X. Zhang, X. Ding, Z. Zhang, C. Bao, L. Guo, L. Chen, X. Tian, *Compos. Sci. Technol.* **2016**, 125, 22.

[21] Z. Zeng, H. Jin, M. Chen, W. Li, L. Zhou, Z. Zhang, *Adv. Funct. Mater.* **2016**, 26, 303.

[22] L. Q. Zhang, S. G. Yang, L. Li, B. Yang, H. D. Huang, D. X. Yan, G. J. Zhong, L. Xu, Z. M. Li, *ACS Appl. Mater. Interf.* **2018**, 10, 40156.

[23] Z. Chen, C. Xu, C. Ma, W. Ren, H. M. Cheng, *Adv. Mater.* **2013**, *25*, 1296.

[24] Sun, X.; Liu, X.; Shen, X.; Wu, Y.; Wang, Z.; Kim, J.-K. *Comp. Part A: Appl. Sci. Manufact.* **2016**, *85*, 199-206.

[25] Shen, B.; Li, Y.; Zhai, W.; Zheng, W. *ACS Appl. Mater. Interf.* **2016**, *8*, 8050.

[26] Y. Wu, Z. Wang, X. Liu, X. Shen, Q. Zheng, Q. Xue, J. K. Kim, *ACS Appl. Mater. Interf.* **2017**, 9 (10), 9059.

[27] W. L. Song, X. T. Guan, L. Z. Fan, W. Q. Cao, C. Y. Wang, M. S. Cao, *Carbon* **2015**, *93*, 151.

[28] C. Liu, S. Ye, J. Feng, *Chem. Asian J.* **2016**, *11*, 2586.

[29] Q. Song, F. Ye, X. Yin, W. Li, H. Li, Y. Liu, K. Li, K. Xie, X. Li, Q. Fu, L. Cheng, L. Zhang, B. Wei, *Adv. Mater.* **2017**, *29*, 1701583.

[30] Y.-J. Wan, P.-L. Zhu, S.-H. Yu, R. Sun, C.-P. Wong, W.-H. Liao, *Carbon* **2017**, 115, 629.

[31] Z. Zeng, C. Wang, Y. Zhang, P. Wang, S. I. Seyed Shahabadi, Y. Pei, M. Chen, X. Lu, *ACS Appl. Mater. Interf.* **2018**, 10, 8205.

[32] O. Pitkanen, J. Tolvanen, I. Szenti, A. Kukovecz, J. Hannu, H. Jantunen, K. Kordas, *ACS Appl. Mater. Interf.* **2019**, 11, 19331.

[33] Z.P., Wu, T. Liu, D.M. Chen, G. Wu, Q. Wang, Y. Yin, Y. Li, Q. Xu, A. Krishamurthy, *RSC Adv.* **2016**, *6*, 62485–62490

[34] T. Kim, D.D.L. Chung, *J. Mater. Eng. Perform.* **2006**, *15*, 295-298.

[35] M. Bayat, H. Yang, F. Ko, D. Michelson, A. Mei, *Polym.* **2014**, *55*, 936-943.

[36] X. Hong, D. D. L. Chung, *Carbon* **2017**, *111*, 529.

[37] C. Wan, J. Li, *Carbhydr. Polym.* **2016**, *150*, 172.

[38] Y. Li, B. Shen, X. Pei, Y. Zhang, D. Yi, W. Zhai, L. Zhang, X. Wei, W. Zheng,

Carbon **2016**, 100, 375.

[39] B. Shen, Y. Li, D. Yi, W. Zhai, X. Wei, W. Zheng, *Carbon* **2016***, 102*, 154.

[40] L. Zhang, M. Liu, S. Roy, E. Chu, K. See, X. Hu, *ACS Appl. Mater. Interf.* **2016**, *8*, 7422.

[41] F. Moglie, D. Micheli, S. Laurenzi, M. Marchetti, V. Mariani Primiani, *Carbon* **2012**, *50*, 1972.

[42] M. Crespo, M. González, A. L. Elías, L. Pulickal Rajukumar, J. Baselga, M. Terrones, J. Pozuelo, *Phys. Stat. Sol. RRL* **2014**, *8*, 698.

[43] A. Gupta, V. Choudhary, *J Mater. Science* **2011**, *46*, 6416.

[44] M. H. Al-Saleh, U. Sundararaj, *Carbon* **2009**, *47*, 1738.

[45] Y. Chen, H.-B. Zhang, Y. Yang, M. Wang, A. Cao, Z.-Z. Yu, *Adv. Funct. Mater.* **2016**, 26, 447.

[46] M. H. Al-Saleh, W. H. Saadeh, U. Sundararaj, *Carbon* **2013**, *60*, 146.

[47] M. Arjmand, M. Mahmoodi, G. A. Gelves, S. Park, U. Sundararaj, *Carbon* **2011**, *49*, 3430.

[48] M. Arjmand, T. Apperley, M. Okoniewski, U. Sundararaj, *Carbon* **2012**, *50*, 5126.

[49] Z. Zeng, M. Chen, H. Jin, W. Li, X. Xue, L. Zhou, Y. Pei, H. Zhang, Z. Zhang, Carbon **2016**, *96*, 768.

[50] Y. Huang, N. Li, Y. Ma, F. Du, F. Li, X. He, X. Lin, H. Gao, Y. Chen, *Carbon* **2007**, *45*, 1614.

[51] Z. Liu, G. Bai, Y. Huang, Y. Ma, F. Du, F. Li, T. Guo, Y. Chen, *Carbon* **2007**, *45*, 821.

[52] S. T. Hsiao, C. C. M. Ma, H. W. Tien, W. H. Liao, Y. S. Wang, S. M. Li, Y. C. Huang, *Carbon* **2013**, *60*, 57.

[53] P. Ghosh, A. Chakrabarti. *Europ. Polym. J.* **2000**, *36*, 1043.

[54] A. A. Eddib, D. D. L. Chung, *Carbon* **2017**, *117*, 427.

[55] K. Ji, H. Zhao, J. Zhang, J. Chen, Z. Dai, *Appl. Surf. Sci.* **2014**, *311*, 351.

[56] T. W. Lee, S. E. Lee, Y. G. Jeong, *ACS Appl. Mater. Interf.* **2016**, 8, 13123.

[57] J. Ma, M. Zhan, K. Wang, *ACS Appl. Mater. Interf.* **2015**, 7, 563.

[58]. Z. Zeng, M. Chen, Y. Pei, S.I. Seyed Shahabadi, B. Che, P. Wang, X. Lu, *ACS Appl. Mater. Interf.* **2017**, *9*, 32211.

[59] Y.-J. Wan, P.-L. Zhu, S.-H. Yu, R. Sun, C.-P. Wong, W.-H. Liao, *Small* **2018**, 14, 1800534.

[60] S. Wu, M. Zou, Z. Li, D. Chen, H. Zhang, Y. Yuan, Y. Pei, A. Cao, *Small* **2018**, *1800634*.

[61] X. Shui, D. D. L. Chung, J. Electr. Mater. **1997**, *26*, 928.

[62] L. Li, D. D. L. Chung, *Composites* **1994**, *25*, 215.

[63] F. Fang, Y.-Q. Li, H.-M. Xiao, N. Hu, S.-Y. Fu, *J.Mater. Chem. C* **2016**, 4, 4193.

[64] Y.-H. Yu, C.-C. M. Ma, C.-C. Teng, Y.-L. Huang, S.-H. Lee, I. Wang, M.-H. Wei, *Mater. Chem.Phys.* **2012**, 136, 334.

[65] M. Arjmand, A. A. Moud, Y. Li, U. Sundararaj, *RSC Adv.* **2015**, 5, 56590.

[66] M. H. Al-Saleh, G. A. Gelves, U. Sundararaj, *Comp. Part A: Appl. Sci. Manufact.* **2011**, 42, 92.
